# Influence of circadian clocks on adaptive immunity and vaccination responses

**DOI:** 10.1038/s41467-023-35979-2

**Published:** 2023-01-30

**Authors:** Louise Madeleine Ince, Coline Barnoud, Lydia Kay Lutes, Robert Pick, Chen Wang, Flore Sinturel, Chien-Sin Chen, Alba de Juan, Jasmin Weber, Stephan J. Holtkamp, Sophia Martina Hergenhan, Jennifer Geddes-McAlister, Stefan Ebner, Paola Fontannaz, Benjamin Meyer, Maria Vono, Stéphane Jemelin, Charna Dibner, Claire-Anne Siegrist, Felix Meissner, Frederik Graw, Christoph Scheiermann

**Affiliations:** 1 Department of Pathology and Immunology, Faculty of Medicine, University of Geneva, Geneva, Switzerland; 2 Department of Medicine, Division of Endocrinology, Diabetes, Nutrition and Patient Education, Faculty of Medicine, University of Geneva, Geneva, Switzerland; 3 Department of Cell Physiology and Metabolism, Faculty of Medicine, University of Geneva, Geneva, Switzerland; 4 Diabetes Center, Faculty of Medicine, University of Geneva, Geneva, Switzerland; 5 Institute of Genetics and Genomics of Geneva (iGE3), University of Geneva, Geneva, Switzerland; 6 Walter-Brendel-Centre of Experimental Medicine, Ludwig-Maximilians-University Munich, BioMedical Centre, Planegg-Martinsried, Germany; 7 Experimental Systems Immunology, Max Planck Institute of Biochemistry, Martinsried, Germany; 8 Systems Immunology and Proteomics, Institute of Innate Immunity, Medical Faculty, University of Bonn, Bonn, Germany; 9 World Health Organization Collaborating Center for Vaccine Immunology, Faculty of Medicine, University of Geneva, Geneva, Switzerland; 10 BioQuant - Center for Quantitative Biology, Heidelberg University, Heidelberg, Germany; 11 Interdisciplinary Center for Scientific Computing, Heidelberg University, Heidelberg, Germany; 12 Geneva Centre for Inflammation Research, Faculty of Medicine, University of Geneva, Geneva, Switzerland; 13 Present Address: Division of Pharmacology & Toxicology, College of Pharmacy, University of Texas at Austin, Austin, TX USA; 14 Present Address: Department of Molecular and Cellular Biology, University of Guelph, Guelph, Ontario Canada

**Keywords:** Circadian rhythms, Adaptive immunity, Conventional dendritic cells, Lymph node

## Abstract

The adaptive immune response is under circadian control, yet, why adaptive immune reactions continue to exhibit circadian changes over long periods of time is unknown. Using a combination of experimental and mathematical modeling approaches, we show here that dendritic cells migrate from the skin to the draining lymph node in a time-of-day-dependent manner, which provides an enhanced likelihood for functional interactions with T cells. Rhythmic expression of TNF in the draining lymph node enhances BMAL1-controlled ICAM-1 expression in high endothelial venules, resulting in lymphocyte infiltration and lymph node expansion. Lymph node cellularity continues to be different for weeks after the initial time-of-day-dependent challenge, which governs the immune response to vaccinations directed against Hepatitis A virus as well as SARS-CoV-2. In this work, we present a mechanistic understanding of the time-of-day dependent development and maintenance of an adaptive immune response, providing a strategy for using time-of-day to optimize vaccination regimes.

## Abstract (web summary)

Circadian rhythms have been shown to influence immune responses, but it is unclear whether this influences responses to vaccines. Here the authors show that dendritic cells migrate in a circadian rhythm meaning that interactions with T cells are altered leading to differential vaccine responses.

## Introduction

Circadian rhythms have emerged as a potent regulator of immune function^1^. Diurnal rhythms have been characterized in multiple aspects of innate immunity, where they can alter the magnitude of immune responses^2–5^. While the concept of rhythmicity in an acute immune response is easier to grasp, the reason why rhythms in adaptive immunity remain intact—even weeks after the initial challenge—has remained elusive, as has the general benefit of a rhythmic immune system for the host.

Despite the long time course of adaptive, relative to innate, immune reactions—weeks compared to days, respectively—it is increasingly apparent that the timing of the initial stimulus can have lasting effects on immune (patho)physiology. Diurnal rhythms in adaptive immune function are seen in the clearance of parasitic worm infections and disease severity in experimental autoimmune encephalomyelitis^6–8^. These discoveries raise the question as to whether diurnal immune rhythms could be used advantageously by administering therapies that target the adaptive immune system at the time of highest sensitivity, such as to elicit enhanced vaccination responses^9^. Temporal variation in antibody production after vaccination has been seen in both humans^10–13^ and mice^14^, but studies of the underlying processes and relative contributions of rhythms in different aspects of the response has been lacking.

To this end, we investigate the mechanisms underlying diurnal rhythms in the generation of adaptive immunity. Using a combination of experimental and mathematical modeling approaches, we investigate how rhythms in cell migration, activation, and effector responses of different cell types interact to generate daily time periods of heightened immune reactivity. Here, we show rhythms at multiple stages of adaptive immune function and propose a model for how these oscillations interact to achieve and maintain temporal variation in response efficacy over long time periods.

## Results

### Day stimulation elicits greater lymph node expansion

**Fig. 1:**
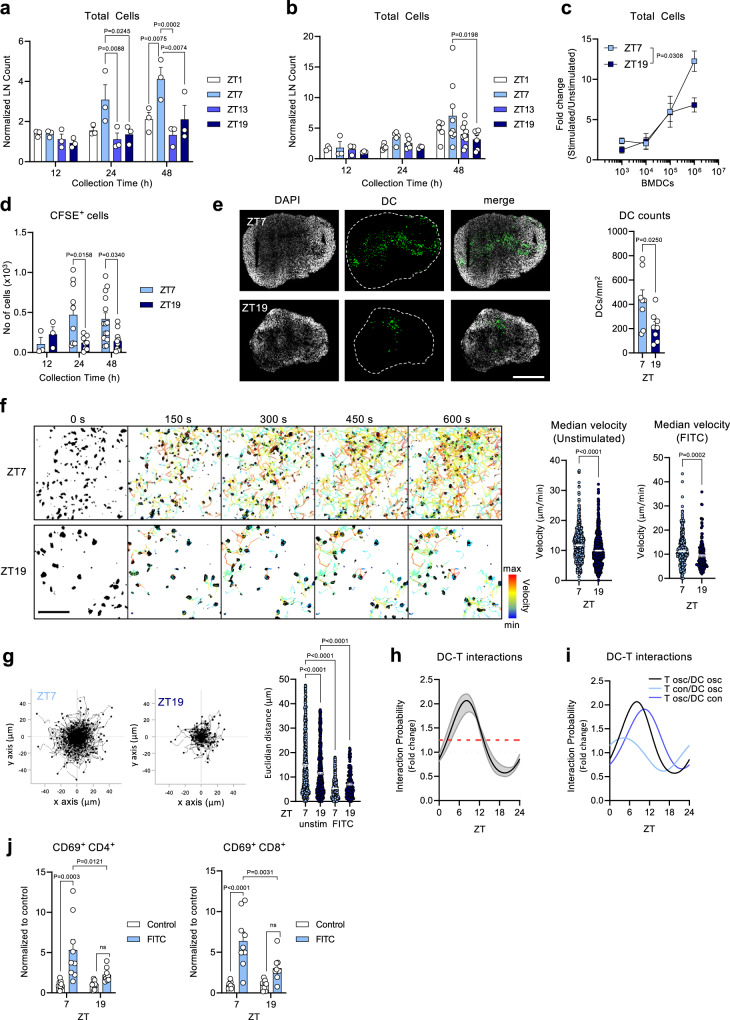
DC migration during the day elicits greater lymph node expansion. **a**, **b** Time course of cell counts, normalized to the 12 h time point on the contralateral side in **a** parotid lymph node (LN), *n* = 3 mice; or **b** popliteal LN, *n* = 3–9 mice; from 5 independent experiments each, two-way ANOVA with Tukey’s post test. **c** Dose-response curve of total cell counts in the popliteal LN 48 h post-injection, normalized to the contralateral side; *n* = 2–3 mice, two-way ANOVA with Sidak’s post test. **d**, **e** Number of exogenous (CFSE^+^) bone marrow-derived dendritic cells (BMDCs) in popliteal LNs quantified by flow cytometry (**d**), *n* = 3–14 mice from 5 independent experiments, two-way ANOVA with Sidak’s post test; or by confocal microscopy (**e**) 24 h post-injection, *n* = 8 mice, unpaired two-sided Student’s t test, scale bar: 500 µm. **f** Median velocities of intravenously injected CD4^+^ T cells in popliteal LN in steady-state conditions and after FITC painting. Tracks were pooled from 3–4 mice per group; *n* = 186–777 total tracks, unpaired two-sided Student’s t test. Scale bar: 50 µm. **g** (Left) Representative overlay of CD4^+^ T cell migration tracks. (Right) Euclidean distance of CD4^+^ T cells from (**f**). Euclidian distances were pooled from 3–4 mice per group; *n* = 186–777 total tracks, one-way ANOVA with Tukey’s post test. **h** Model predictions (Supplementary Table 1 (ID1, ID2)) for the fold-change in the interaction probability of DC and T cells comparing rhythmic migratory dynamics (red dashed line) to non-rhythmic migration (gray dotted line). The mean (black line) and the range (min–max, gray shaded area) of the predicted fold change across skin draining LNs are shown. **i** Model predictions for the fold-change in the interaction probability of DC and T cells, assuming different combinations of rhythmic and non-rhythmic components based on the mean dynamics across skin draining LNs. **j** Number of CD69^+^ CD4^+^ and CD8^+^ T cells in draining LNs 48 h post-FITC; *n* = 9 mice; two-way ANOVA with Tukey’s post test. Data are plotted as mean ± standard error of mean (SEM); ns, not significant. Source data are provided as a Source data file and detailed sample sizes are available in “Methods”.

To investigate why adaptive immune responses are time-of-day dependent, we focused on the mouse skin as one of the epithelial surfaces for antigen encounter and thus a primary site for the initiation of immune responses. We painted mouse ears with fluorescein isothiocyanate (FITC) at four different times across the day and quantified the ensuing endogenous response in the draining parotid lymph node (LN). Total LN cellularity increased much more strongly when ears were painted in the afternoon (at *Zeitgeber* time 7, i.e., 7 h after light onset in a 12 h:12 h light:dark environment) compared to all other times, with differences observed starting 24 h after stimulation (Fig. 1a and Supplementary Fig. 1a).

To investigate the validity of these time-of-day differences, we assessed whether rhythmicity in the response also applied to another stimulus at a different skin site. Exogenous LPS-activated bone marrow-derived dendritic cells (BMDCs) were subcutaneously injected into the hock of the leg and the response was quantified in the draining popliteal LN. This experimental setup further allowed us to better control for potential differences in numbers and activation status of skin residing leukocytes, since we administered the same batch of BMDCs injected into phase-shifted recipient mice that were housed under shifted lighting regimes. This ensured microenvironmental timing and not timing in the DCs to be the only variable. These experiments yielded a similar rhythmicity in the expansion of the draining LN compared to FITC-painting (Fig. 1b and Supplementary Fig. 1b–d). This demonstrated draining LN responses to be highly time-of-day dependent, even with respect to different stimuli elicited at distinct skin sites. Analyses of the cell populations within the LN showed that CD4^+^ and CD8^+^ T cell numbers as well as B cells increased strongly after stimulation during the day in contrast to the night, representing the predominant leukocyte subsets, while overall LN composition did not change (Supplementary Fig. 1e–g). Notably, titration of the amount of injected exogenous BMDCs showed ZT19 (night) injection of 10^6^ cells to exhibit similar efficacy as injection of ten times fewer cells, while ZT7 (day) injection still showed increased efficacy (Fig. 1c and Supplementary Fig. 1g). This demonstrated that the time-of-day effect could not be overcome simply by injecting a higher number of BMDCs, indicating that a plateau effect had been reached during the night phase in these assays.

### Dendritic cells migrate better to the LN during the day

Since in these experiments of BMDC injection, timing in the recipient was the only variable, our results indicated that the microenvironment governed the response, potentially by affecting the migration kinetics of BMDCs and/or by shaping their phenotype^15–17^. While we did not detect changes in the phenotype of donor BMDCs recovered from the draining LN (Supplementary Fig. 2a, b), we observed a pronounced time-of-day difference with respect to their numbers, with more BMDCs having drained to the LN when experiments were performed during the day (Fig. 1d, e). There was no change with respect to the location of injected cells after 24 h; at both time points, BMDCs were found to predominantly locate to the T cell area of the LN (Supplementary Fig. 2c, d). These data demonstrate migration of dendritic cells (DCs) from the skin to the draining LN to occur in a diurnal manner with a strong influence on the level of LN expansion, providing a functional relevance for the temporal migration phenotype of DCs into afferent skin lymphatics recently described^18^.

Using 2-photon intravital microscopy (2P-IVM) we analyzed the migration of adoptively transferred T cells within the draining LN at the single-cell level and detected an increased velocity during the day, both in steady-state conditions and after FITC painting (Fig. 1f, g). Building on our previous data with respect to circadian rhythms in T cell trafficking to LNs^7^ (Supplementary Fig. 3, Supplementary Table 1), we developed a mathematical model to combine these dynamics in LN infiltration and migration velocity with the observed rhythmic DC migration from the skin to the draining LN (Supplementary Fig. 4, Supplementary Table 1). This allowed us to quantitatively assess the probability of LN encounters between DCs and T cells across a diurnal cycle, generating an oscillatory likelihood in their interactions, with a peak occurring in the afternoon (Fig. 1h). This indicated that in-phase migration of DCs from skin epithelial barrier sites and infiltration of T cells from blood to the draining LNs would be beneficial for the encounter of the respective antigen-bearing and antigen-recognizing cells, with a ~2-fold higher interaction probability compared to constant, non-rhythmic leukocyte trafficking behavior. In contrast, simulations that rendered LN immigration of DCs and/or T cells arrhythmic reduced the amplitude in the likelihood of DC-T cell interactions (Fig. 1i).

To directly validate these predictions experimentally, we measured T cell activation after FITC painting using the surface marker CD69 as proxy. When the stimulus occurred during the day, the amount of CD69^+^ CD4^+^ and CD8^+^ T cells was significantly higher 48 h after FITC application, compared to nighttime (Fig. 1j and Supplementary Fig. 5a). Together, this demonstrates a benefit of circadian rhythmicity in adaptive immunity to the host as the probability for functional encounters between DCs and cognate T cells is increased when both cell types enter LNs and are present in this tissue around the same time of day.

### Rhythmic DC draining induces lymph node homing

**Fig. 2:**
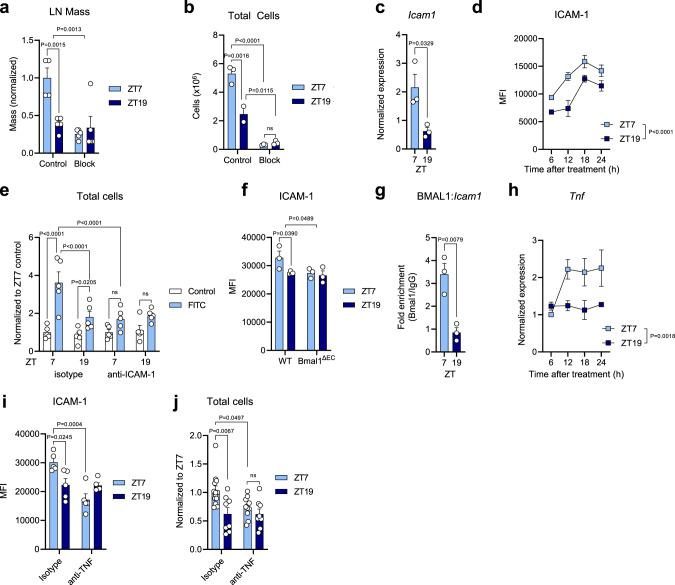
Rhythmic DC trafficking induces lymph node homing. **a** Popliteal lymph node (LN) mass 24 h following subcutaneous injection of 1 × 10^6^ bone marrow-derived dendritic cells (BMDCs), with or without prior treatment with integrin-blocking antibodies, normalized to average ZT7 mass; *n* = 4–5 mice, two-way ANOVA with Sidak’s post test. **b** Popliteal LN cellularity 48 h following subcutaneous injection of 1 × 10^6^ BMDCs, with and without prior treatment with integrin-blocking antibodies; *n* = 2–3 mice, two-way ANOVA with Sidak’s post test. **c** *Icam1* mRNA expression in parotid LN 12 h after topical application of FITC; *n* = 3 mice, unpaired two-sided Student’s t test. **d** Time course of ICAM-1 protein levels on high endothelial venules (HEVs) of parotid LNs by quantitative imaging after topical FITC application; *n* = 3 mice, two-way ANOVA with Sidak’s post test. **e** LN cellularity 48 h after topical FITC application, with or without prior treatment with anti-ICAM1 antibody; *n* = 5 mice, two-way ANOVA with Fisher’s post test. **f** ICAM-1 protein levels on HEVs of parotid LNs 12 h after topical FITC application in WT or endothelial cell-specific *Bmal1*^*−/−*^ mice (BMAL1^ΔEC^); *n* = 3–4 mice, two-way ANOVA with Sidak’s post test. **g** Chromatin immunoprecipitation (ChIP) analysis of BMAL1 binding to the *Icam1* promoter region R4 in the parotid LN 6 h after topical FITC application; *n* = 3 mice, unpaired two-sided Student’s t test. **h** Time course of *Tnf* mRNA expression in parotid LN after topical application of FITC; *n* = 3 mice, two-way ANOVA with Sidak’s post test. **i** ICAM-1 protein levels on HEVs of parotid LNs 12 h after topical FITC application in mice pre-treated with anti-TNF antibody or isotype control; *n* = 5 mice, two-way ANOVA with Tukey’s post test. **j** Parotid LN cellularity 48 h after topical FITC application, with or without prior treatment with anti-TNF antibody; *n* = 8–14 mice, two-way ANOVA with Tukey’s post test. Data are plotted as mean ± standard error of mean (SEM); ns, not significant. Source data are provided as a Source data file and detailed sample sizes are available in “Methods”.

Since increased LN cell counts occurred as early as 24 h after injection, this phenotype was not caused by leukocyte proliferation (Supplementary Fig. 5b) but instead pointed to altered leukocyte homing from blood. Indeed, blocking leukocyte infiltration with antibodies directed against the LN homing receptors α_4_ (CD49d) and α_L_ (CD11a) integrins completely abrogated the increase in LN mass and cellularity. This block also rendered leukocyte LN counts arrhythmic (Fig. 2a, b), without influencing BMDC draining from the skin (Supplementary Fig. 5c). Thus, the time-of-day-dependent increase in LN cellularity resulted from homing of cells from blood.

Loss of rhythmic LN cellularity upon ablation of integrin function indicated that migration of DCs to the LN at specific times of the day rendered the latter more permissive for leukocyte infiltration from blood. Indeed, we detected higher mRNA levels of *Icam1*, the β2 integrin counter receptor and critical homing molecule for LN trafficking in inflammation^19^, in the LN after stimulation during the day compared to the night (Fig. 2c). We further observed increased ICAM-1 protein expression by quantitative imaging at this time, specifically in high endothelial venules (HEVs), the sites of leukocyte infiltration into the LN (Fig. 2d and Supplementary Fig. 5d). ICAM-1 was functionally involved in the recruitment phenotype, since treatment with an antibody directed against ICAM-1 reduced LN expansion and abrogated the time-of-day differences (Fig. 2e). Interestingly, the *Icam1* gene exhibits several canonical and non-canonical E-box binding sites for the core circadian transcription factor BMAL1, indicating the potential for direct control by the circadian clock machinery (Supplementary Fig. 5e). Indeed, *Cdh5*^*CreERT2*^*:Bmal1*^*flox*^ mice, exhibiting an inducible deficiency in endothelial cell BMAL1 (BMAL1^ΔEC^), failed to rhythmically upregulate ICAM-1 expression in HEVs in this inflammatory scenario, demonstrating a role for the endothelial circadian clock in driving time-of-day-differences in ICAM-1 expression (Fig. 2f). In line with these data, chromatin immunoprecipitation (ChIP) assays of LNs after FITC painting demonstrated BMAL1 to directly bind the canonical E-box regions R4 (and to a lesser extent R3) in the *Icam1* promoter, and this interaction exhibited rhythmic occupancy, being higher after stimulation during the day than at night (Fig. 2g and Supplementary Fig. 5f). To further investigate the mechanism by which DC drainage to the LN would increase ICAM-1 expression^15^, we quantified levels of pro-inflammatory cytokines in draining LNs. Q-PCR analyses showed *Tnf* levels to be rhythmically expressed in LNs after FITC painting—but not of other cytokines or chemokines (Fig. 2h and Supplementary Fig. 6a–c). Furthermore, blocking TNF with an anti-TNF antibody downregulated ICAM-1 expression and rendered it arrhythmic (Fig. 2i). This treatment furthermore reduced LN expansion and abrogated the time-of-day differences (Fig. 2j). These data demonstrate rhythmic draining of DCs into the LN to induce a time-of-day gated infiltration of leukocytes from the blood. This LN expansion is functionally associated with an increase in TNF and ensuing ICAM-1 expression in HEV, the latter of which represents a phenomenon directly controlled by the endothelial cell circadian clock machinery.

### CD4^+^ T cell autonomous circadian rhythms in immune responses

**Fig. 3:**
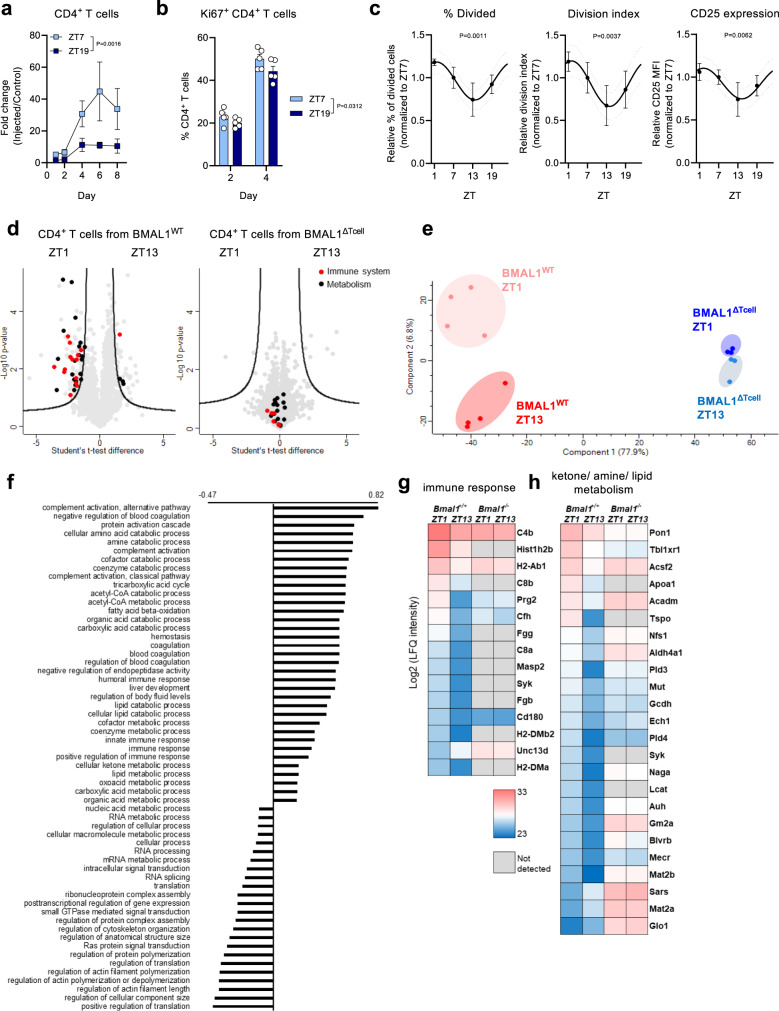
CD4^+^ T cell-intrinsic, clock-controlled rhythms in the adaptive immune response. **a** Time course of CD4^+^ T cells in popliteal lymph nodes (LNs) following subcutaneous injection of 1 × 10^6^ bone marrow-derived dendritic cells (BMDCs); *n* = 5 mice, two-way ANOVA with Sidak’s post test. **b** Quantification of Ki67^+^ CD4 T cells in popliteal LNs following subcutaneous injection of 1 × 10^6^ BMDCs; *n* = 5 mice, two-way ANOVA with Sidak’s post test. **c** Proliferation capacity of LN CD4^+^ T cells stimulated ex vivo, normalized to the ZT7 average; *n* = 3–6 mice, cosinor analysis (F-test with 95% confidence interval; 15 degrees of freedom; R^2^ effect sizes from left to right 0.5949, 0.5261, 0.4918). **d** Volcano plot of protein enrichment in LN CD4^+^ T cells from WT (left) and T cell-specific *Bmal1*^*−/−*^ (right, (BMAL1^ΔTcell^)) mice in steady state at ZT1 vs. ZT13; *n* = 3–4 mice, unpaired two-sided Student’s t-test, ZT1 vs. ZT13 at a FDR = 0.05 and S0 = 1 that are part of the GOBP terms immune response (red) or ketone/amine/lipid metabolism (black) are shown. **e** Principal component analysis of log_2_ transformed LFQ intensities at a FDR = 0.05 cut-off. **f** GOBP 1D annotation categories and enrichment scores of the unpaired two-sided Student’s t-test differences ZT1 vs. ZT13 at a FDR = 0.01 cut-off. **g**, **h** Heatmap of log_2_ transformed LFQ intensities filtered for significance in a Student’s t-test ZT1 vs. ZT13 and part of the GOBP terms immune response (**g**) or ketone/amine/lipid metabolism (**h**). Data are plotted as mean ± standard error of mean (SEM) ns, not significant. Source data are provided as a Source data file and detailed sample sizes are available in “Methods”.

While these data demonstrated rhythmicity in the acute, initiation phase of an adaptive immune response in the LN, we next extended our analyses to the ensuing week to assess whether and how rhythmicity was maintained. LN CD4^+^ T cell counts continued to exhibit time-of-day differences well past the initiation phase of the immune response (Fig. 3a). Furthermore, we observed differences in their proliferation rates, indicating that local proliferation—in addition to homing—was also regulated in a circadian manner^9,20^, and could contribute to the differences at later time points in this setting (Fig. 3b). Indeed, in a reductionist in vitro scenario, CD4^+^ T cells isolated from the LN at different times of the day exhibited stronger proliferation capacities during the day than at night (Fig. 3c). This demonstrated CD4^+^ T cell proliferation as a cell-intrinsic mechanism contributing to circadian changes in LN cellularity.

To obtain a global overview of the time-of-day-dependent changes responsible for this effect, we performed proteomics analyses by mass spectrometry of highly purified CD4^+^ T cells isolated from the LN during the day peak (ZT1) and night trough (ZT13). Our results indicated CD4^+^ T cells to exhibit a strong difference in the time-of-day-dependent protein expression profile (Fig. 3d–f). Specifically, an enhanced lipid, ketone or amine metabolic and immune regulation signature was observed in the morning, in line with the proliferation differences (Fig. 3f). Comparing these temporal proteomics data with CD4^+^ T cells isolated from T cell specific *Bmal1*^*−/−*^ mice (BMAL1^ΔTcell^) showed a strikingly different protein expression profile, with strongly reduced expression of rhythmic proteins in the latter and only few molecules exhibiting diurnal differences (Fig. 3d–h). Immune regulation and metabolism-related differences were lost in BMAL1 deficiency, resulting in an almost indistinguishable proteome between early and late times (Fig. 3e). Accordingly, numerous proteins belonging to the immune regulation and metabolism clusters were either not detected in BMAL1^ΔTcells^ (Fig. 3g) or displayed no diurnal differences (Fig. 3h). This demonstrated that CD4^+^ T-cell autonomous rhythms in metabolism and proliferation exhibit time-of-day differences, contributing to the functional time-of-day differences in the immune response.

### Co-ordinated cell-intrinsic oscillators maintain rhythmicity

**Fig. 4:**
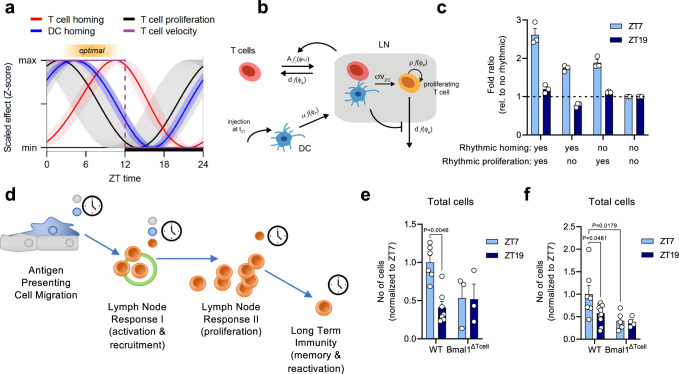
Modeling a multi-stage process for adaptive immunity by integrating cell-intrinsic rhythms. **a** Dynamics of the individual rhythmic components of T cell (red) and dendritic cells (DCs, blue) migration to the lymph node (LN), as well as T cell proliferation (black) as predicted by the mathematical models (ID1-3, Supplementary Table 1) with Z-score for each dynamic being shown. The solid lines indicate the mean (for T cell homing) or the best fit (for DC homing and T cell proliferation), while colored shaded areas represent 95%-confidence intervals. T cell velocity (purple) was only measured at ZT7 and ZT19, leading to a stepwise function. The orange shaded area indicates a time window for potential optimal interactions of rhythmic components around ZT7. **b** Mathematical model (Supplementary Note 1) describing the migration and interaction of T cells and DC to and within the LN. T-DC interactions lead to activated T cells (orange) that proliferate in a rhythmic manner, and mediate feedback on the homing and egress dynamics of T cells. **c** Predicted effect of individually ablating rhythmic homing (T + DC) or proliferation (T) on LN expansion at day 6 post-injection relative to an arrhythmic scenario. Individual data points show predicted fold ratio for each skin draining LNs using the best fit, with bars and whiskers indicating the mean ± 1.96 × SEM, *n* = 3 mice. **d** Schematic depicting stages of initial immune response and clock interactions. Antigen-presenting cell (APCs) trafficking is regulated by clocks in endothelial cells (green) and APCs (blue), leading to temporal variation in APC numbers reaching the LN. This feeds into rhythms in the acute LN response to influence recruitment dynamics of effector cells (red). Downstream and independent of these rhythmic events, effector cells can maintain a rhythmic proliferation capacity, ultimately leading to temporal variation in long-term immunity. **e**, **f** Leukocyte counts 48 h post-treatment in WT and T cell-specific *Bmal1*^*−/−*^ (BMAL1^ΔTcell^) mice in **e** parotid LNs, *n* = 3–7 mice; and **f** popliteal LNs, *n* = 4–11 mice; two-way ANOVA with Tukey’s post test. Data are plotted as mean ± standard error of mean (SEM); ns, not significant. Source data are provided as a Source data file and detailed sample sizes are available in “Methods”.

We next integrated the oscillatory dynamics of the individual rhythmic components of T cell and DC homing to the LN, as well as T cell proliferation and velocity in the LN to assess how these factors might interact to create enhanced immune responsiveness at specific times. Mathematical modeling identified optimal conditions for LN expansion within a time window located to the afternoon, confirming our experimental observations (Fig. 4a, Supplementary Figs. 3–4 and 7, Supplementary Table 1). We extended our mathematical model on the likelihood of rhythmic DC-T cell interactions in the LN (Fig. 1h, i) to assess the effect of the interplay of the various rhythmic components on LN expansion and the required conditions for the immune response to remain rhythmic for several weeks after challenge (Fig. 4b, Supplementary Note 1 and Supplementary Table 1). Combining the acute rhythmic infiltration of lymphocytes and their proliferation allowed us to demonstrate that both homing and proliferation were required to obtain rhythmicity in LN expansion (Fig. 4c). Simulations where rhythmicity was lost in either of the components led to reduced LN expansion and ablated overall time-of-day differences (Fig. 4c). This validated our findings that multiple, separate but interacting steps were building onto each other to maintain the rhythmicity observed early on to longer time periods (Fig. 4d and Supplementary Note 1). This also indicated that rhythmicity not only in single cellular subsets but across multiple cell types was required and that lack of rhythm in any one of these components would abrogate overall oscillations. To validate our model predictions, we performed experimental analyses of LN cellularity after FITC painting or BMDC injection using BMAL1^ΔTcell^ recipients (Fig. 4e, f). In both scenarios, total cellularity of the LN was no longer rhythmic in conditions of BMAL1 deficiency, demonstrating the importance of the T cell circadian clock machinery in this process.

### Relevance of rhythmic adaptive immunity in vaccination

**Fig. 5:**
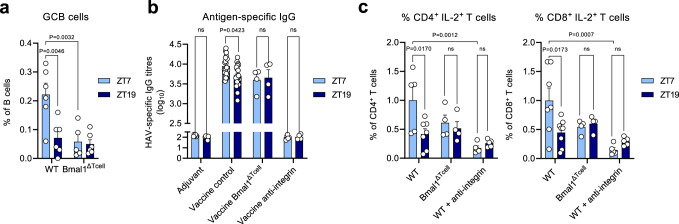
Rhythms in response to vaccination. **a** Germinal center B (GCB) cells as % of B cell fraction in the draining inguinal LN of T cell specific *Bmal1*^*−/−*^ (BMAL1^ΔTcell^) mice 14 days following vaccination with the commercial vaccine HAVRIX; *n* = 5–6 mice, two-way ANOVA with Tukey’s post test. **b** Serum antigen-specific antibody titers 28 days after vaccination with the commercial vaccine HAVRIX; *n* = 4–22 mice, two-way ANOVA with Tukey’s post test. **c** T cell response upon antigen restimulation 28 days after vaccination with the commercial HAVRIX vaccine; *n* = 4–8 mice, two-way ANOVA with Tukey’s post test. Data are plotted as mean ± standard error of mean (SEM); ns, not significant. Source data are provided as a Source data file and detailed sample sizes are available in “Methods”.

To investigate whether these initial time-of-day-differences could yield long-lasting, potentially clinically relevant effects, we employed a vaccination model against Hepatitis A virus (HAV). Vaccination of mice with a commercial human HAV vaccine (HAVRIX) during the day with a single dose resulted in significantly enhanced germinal center B (GCB) cell numbers 14 days after vaccination compared to nighttime (Fig. 5a and Supplementary Fig. 8a). In contrast, and in agreement with our earlier observations, BMAL1^ΔTcell^ mice showed strongly reduced and non-rhythmic GCB levels (Fig. 5a). Importantly, the diurnal differences translated to enhanced anti-HAV antibodies in serum at day 28, illustrating that initial timing of vaccination could be an effective means of enhancing antibody titers (Fig. 5b). In addition to humoral immunity, we also observed time-of-day differences in the T cell response. LN were harvested 28 days after HAV vaccination and re-stimulated with HAV antigen to quantify antigen-specific T cell responses. We observed higher percentages of IL-2^+^ CD4^+^ and CD8^+^ T cells when vaccination had been performed at ZT7 compared to ZT19 (Fig. 5c). Furthermore, time-of-day differences were abrogated in BMAL1^ΔTcell^ mice (Fig. 5b, c). In addition, blocking leukocyte homing with anti-integrin antibodies abrogated time-of-day differences and reduced overall antibody titers and T cell responses (Fig. 5b, c) in line with our previous observations (Fig. 2a, b). We further investigated whether this time-of-day difference in vaccination would also apply to SARS-CoV-2 (severe acute respiratory syndrome coronavirus type 2), where optimization of vaccine doses is particularly critical. We employed a vaccination model against SARS-CoV-2, using its spike RBD protein. LN were harvested 28 days after challenge and re-stimulated with RBD to quantify antigen-specific T cell responses. Both CD4^+^ and CD8^+^ T cell responses were significantly stronger when the initial vaccination had been performed during the day, with respect to quantity of IL-2 production and percentage of IL-2-producing cells, as well as IL-4 and IL-17 levels for CD4^+^ T cells (Supplementary Fig. 8b, c). Together, these data demonstrate that both humoral and T cell-mediated adaptive immune responses maintain their rhythmic differences, even 28 days after the initial time-of-day dependent stimulation.

## Discussion

We show here that DCs enter the draining LN in a time-of-day-dependent manner, with a rhythmic adaptive immune response as consequence. We observed this phenomenon after FITC-painting, which activates both endogenous DCs as well as the tissue microenvironment, as well as after injection of non-synchronized BMDCs, a scenario where the influence of time-of-day is only assessed in the microenvironment and not in the DCs. We found rhythmicity to be initially governed by homing of cells from the blood due to TNF and endothelial cell BMAL1-controlled expression of ICAM-1 on HEVs. These oscillations are beneficial to the host as rhythmic presence of antigen-presenting DCs and antigen-recognizing T cells in the draining LN provides an enhanced likelihood in their functional encounters. We show that the response in the early days continues into the first week where now proliferation responses are seen contributing to the cellularity increase, themselves also being under control of the circadian clock. These time-sensitive response patterns extend further to enhanced germinal center formation after 14 days and enhanced antibody titers and T cell responses 28 days after an initial vaccination with a single dose. Thus, by tracing the generation of an adaptive immune response over several weeks, we observed rhythmicity in every step investigated, with the timing of initial challenge imprinted on all downstream processes. Developing a mechanistic framework for the interaction of the individual processes showed the importance of this interaction, as presence of rhythmicity in each component provided advanced efficacy in the optimal time window compared to scenarios where rhythms were lost. Thus, the circadian clock appears to regulate and maintain an adaptive immune response at a physiological level over long time frames, preventing it from both overreaction and decline. Enhanced immune reactivity just after the middle of the rest phase and before the onset of the behavioral activity phase may prime the host to an enhanced likelihood to encounter pathogens, such as occurring during the search for food or due to social interactions.

This work is in line with evidence showing regulation of adaptive immune responses by time-of-day and endogenous circadian rhythms^4,7,9,14^ and provides mechanistic insights into a diurnal regulation of vaccination responses. Previous research has highlighted the immune-enhancing effects of sleep upon Hepatitis A and B vaccination^11,12,21^, and established a link between vaccination timing and antibody production in Influenza^13^. Furthermore, time-of-day was recently shown to influence off-target trained immunity after anti-tuberculosis vaccination with bacillus Calmette-Guérin (BCG), suggesting that optimization of vaccination timing may also lead to much broader immunological benefits by providing a stronger training signal for immunity in the morning than in the evening^10^. This optimal time window appears to be consistently situated around or just before the behavioral activity phase across human and rodents and using different stimuli, likely due to the common underlying mechanisms of immune cell trafficking, proliferation, and germinal center formation we report here. While for nocturnal mice this optimal window appears to be the afternoon, in diurnal humans this may be the early morning.

Since we see rhythmicity preserved in vaccination responses with a single dose, our findings are of direct public health relevance^22^. Particularly in cases of limited vaccine availability or an urgent need for high levels of protective immunity, as currently observed in the SARS-CoV-2 pandemic, efficiently exploiting rhythmicity could optimize vaccination regimes^23^. Given that human and mouse rhythms exhibit analogous peaks and troughs in their respective behavioral activity and rest phases^24,25^, it would thus be important for clinical studies to include and report time-of-day as a critical variable for further investigations and potential improvement of treatment efficacy.

## Methods

### Ethics statement

All animal procedures and experiments were performed in strict accordance with all mandatory guidelines (EU and Swiss directives on the protection of animals used for scientific purposes), approved by the ministry of animal welfare of the region of Oberbayern and performed in accordance with the German law of animal welfare or approved and performed in accordance with the guidelines of the animal research committee of Geneva (Commission Cantonale pour les Expériences sur les Animaux (CCEA) and the Office fédéral de la santé alimentaire et des affaires vétérinaires (OSAV)).

### Animals

C57BL/6N wild-type (WT) mice (*Mus musculus*, mixed sexes, 7–12 weeks old) were purchased from Charles River. The following transgenic mice were cross-bred at ENVIGO to yield T cell-specific (BMAL1^ΔTcell^, *Mus musculus*, C57BL/6 background, mixed genders, 7–12 weeks old) and inducible endothelial cell specific (BMAL1^ΔEC^ *Mus musculus*, C57BL/6 background, mixed sexes, 5–12 weeks old) *Bmal1*-deficient mice: *Bmal1*^*flox/flox*^, *Cd4*^*Cre*^ (both purchased from Jackson labs), *Cdh5*^*CreERT2*^ (gift from Dr. Ralf Adams, Max-Planck-Institute for Molecular Biomedicine Münster, Germany). Primer sequences are provided in Supplementary Table 2. Mice were housed under a 12 h:12 h light:dark schedule, with temperature maintained between 20–22 °C and a humidity level around 50%. Mice had access to food and water ad libitum. Prior to experiments, recombination was induced in the BMAL1^ΔEC^ line via intraperitoneal injection of tamoxifen on 5 consecutive days. Where multiple time points were investigated simultaneously, light-tight cabinets (Tecniplast) were used to shift animals to the respective phase a minimum of 1 day/1 h shift prior to the experiments. Treatment times correspond to *Zeitgeber* time (ZT) and indicate timing relative to lights on in the animal facility such that ZT1 is 1 h after lights on (morning), ZT7 is 7 h after lights on (day time), ZT13 is 1 h after lights off (evening) and ZT19 is 7 h after lights off (night time).

### Reagents and resource sharing

Commercial reagents and kits, as well as antibodies used in this study are listed in Supplementary Tables 3 and 4, respectively.

### Subcutaneous injection of bone marrow-derived dendritic cells

Bone marrow-derived dendritic cells (BMDCs) were cultured as previously described^26^, with complete culture media (RPMI, 10% FCS, 2 mM L-glutamine, 1% penicillin & streptomycin, 50 µM β-mercaptoethanol) supplemented with 20 ng/ml recombinant mouse GM-CSF. At day 10, all non- and semi-adherent cells were transferred to a 6 cm dish in complete media with 10 ng/ml GM-CSF and stimulated with 100 ng/ml lipopolysaccharide (LPS) for 24 h. Following stimulation, cells were counted, adjusted to a concentration of 10 × 10^6^ cells/ml in PBS, and stained with a final concentration of 3 µM CFSE at 37 °C for 20 min with gentle agitation. Following staining, cells were washed twice with complete media and resuspended at 50 × 10^6^ cells/ml in PBS. Mice were anesthetized by isoflurane inhalation and 20 µl (1 × 10^6^ cells) of this solution was injected subcutaneously into the hock of each mouse. For the dose-response experiments, the cell solution was diluted with PBS to maintain the same injection volume.

### Topical FITC stimulation

Fluorescein isothiocyanate (FITC) powder was weighed and diluted to a concentration of 8 mg/ml in a 1:1 mixture of dibutyl phthalate and acetone. The mixture was thoroughly vortexed prior to use. Mice were anesthetized by isoflurane inhalation and 25 µl of this solution was applied to the ear skin.

### Flow cytometry

Samples were collected at the indicated time points following stimulation. In order to obtain single-cell suspensions, organs were first finely minced with scissors and incubated in 1 ml digestion mix (PBS with Ca^2+^ and Mg^2+^, 1 mg/ml collagenase IV, and 0.2 mg/ml DNase I) for 30 min at 37 °C under constant agitation. For analysis of germinal centers following vaccination, digestion was unnecessary, and this step was omitted. Samples were processed through 70 µm cell strainers (Corning) with an excess volume of PEB (PBS with 1% BSA and 2 mM EDTA) and centrifuged at 300 g for 5 min. For spleen samples, cell pellets were resuspended in 5 ml RBC lysis buffer (155 mM NH_4_Cl, 0.1 mM KHCO_3_, 0.1 mM EDTA in ddH_2_O) and incubated for 5 min at room temperature. 5 ml PEB was added to stop the lysis reaction and samples were centrifuged again. Cell pellets were resuspended in PEB prior to staining. Spleen samples were counted and 2 × 10^6^ cells were taken for staining to ensure saturating conditions. LN samples were stained in their entirety with a small aliquot taken for counting during staining on a Coulter Counter (Z2, Beckman Coulter; Countess II, Invitrogen).

For all staining panels, an initial blocking step was performed to reduce non-specific binding. Cells were incubated with 10 µg/ml purified rat anti-mouse CD16/32 antibody in PEB at room temperature for 5–15 min. Following this incubation, an equivalent volume of 2x concentrated antibody staining mix was added directly to the cell suspension and samples were incubated for a further 30 min at 4 °C. Antibodies used for immunostaining are listed in Supplementary Table 4.

Cells were washed and resuspended in 300 µl PEB with assay-dependent viability dye (DAPI, Propidium Iodide, or DRAQ7) and characterized using a 10-color Gallios (Beckman Coulter) or 18-color BD LSR Fortessa (BD Biosciences) flow cytometer. Acquired data was analyzed using Kaluza (Beckman Coulter), FACSDiva 8 (BD Biosciences), and FlowJo 10 (TreeStar) software. Debris was excluded based on forward and side scatter profiles, and viability dye was used to remove dead cells. Fluorescence minus one (FMO) controls were used to define gate positions. Differential cell counts were obtained by multiplying total counts (Coulter Counter Z2, Beckman Coulter; Countess II, Invitrogen) by the gated frequencies, or by using CountBright Absolute Counting Beads during acquisition.

### Q-PCR

Samples were collected at the indicated time points and immediately frozen on dry ice and stored at −80 °C. Tissues were homogenized in Trizol using a Precellys 24 (Bertin) bead mill homogeniser and CK14 tubes (Bertin). Lysed and homogenized samples were then processed for RNA extraction using a Direct-zol RNA MiniPrep kit according to manufacturer’s instructions. RNA quantity and quality were analyzed by Nanodrop 2000 (ThermoFisher) and 200 ng was taken for cDNA synthesis. Reverse transcription was performed with High-Capacity RNA-to-cDNA Kit and resulting cDNA was diluted to 1 ng/µl. Q-PCR analyses were performed using PowerUp SYBR Green (Applied Biosystems): primer sequences are provided in Supplementary Table 2. Quantification of transcript was performed with StepOne v2.3 software using the 2^-ΔΔCt^ method using *Rpl32* as an internal reference gene.

### In vivo antibody treatments

To block homing of blood leukocytes, integrin-blocking antibodies (anti-mouse CD49d, and anti-mouse CD11a, Supplementary Table 4) were injected i.p. at a concentration of 100 µg/mouse 6 h prior to BMDC injection. To block ICAM-1, anti-mouse ICAM-1 was injected i.p. at a concentration of 100 µg/mouse at the time of FITC treatment. To block TNF, anti-mouse TNF was injected i.p. at a concentration of 100 or 500 µg/mouse at the time of FITC treatment. For isotype control, we combined Rat IgG2b isotype and anti-horseradish peroxidase at a concentration of 50 µg/mouse each and injected i.p. together at the time of FITC application.

### Immunofluorescence imaging of lymph node sections

LNs were collected 24 h after BMDC injection, embedded in cryomolds using OCT Compound, and stored at −80 °C. 10 µm sections were cut using a cryostat (Leica), with slices collected onto poly-l-lysine coated glass slides. Sections were encircled with a hydrophobic pen prior to staining, and fixed with ice-cold methanol for 10 min. Slides were then washed with PBS, and incubated in blocking solution (PBS with 0.5% Triton X-100 and 20% normal goat serum) for 1 h at room temperature. Following the blocking step, slides were washed and a fluorescent antibody mix was applied. For analysis of BMDC migration, 2 µg/ml anti-mouse CD31-APC and DAPI, diluted in blocking buffer, were used as counterstains. For analysis of ICAM-1 expression, anti-mouse CD31-APC and anti-mouse ICAM-1-PE or its corresponding Rat IgG2b,κ isotype control, diluted in blocking buffer, were used as counterstains. Slides were incubated overnight at 4 °C in the dark, and then washed in PBS prior to imaging. Images were acquired using an Axio Examiner.Z1 spinning disk confocal microscope (Zeiss) equipped with 405, 488, 561, and 640 nm laser sources and a 20× objective using Slidebook 6 software (Intelligent Imaging Innovations). Cell quantification was performed using ImageJ (version 1.51n, FIJI). Migration velocity and Euclidean distance were analyzed offline using ImageJ software (NIH, USA) and the tracking plugin TrackMate. Single-cell migration tracks were generated using chemotaxis and migration software provided by Ibidi (Germany).

### Two-photon intravital microscopy of the lymph node

For each experiment, donors and recipients were maintained in the same circadian phase. Donor mouse spleens were harvested 6 h prior to imaging time (i.e., ZT1 for the ZT7 imaging experiments and ZT13 for the ZT19 imaging experiments, to provide an experimental setup in the most physiological context, i.e., timing in the T cell and the lymph node) and homogenized by passing through a 40 µm cell strainer (Corning). After flushing with PEB (PBS supplemented with 1% bovine serum albumin and 2 mM EDTA), the sample was centrifuged at 300 × *g* for 5 min. The pellet was resuspended in 1 ml PEB and CD4^+^ T cells were isolated by negative selection using the EasySep^TM^ Mouse CD4^+^ T Cell Isolation Kit according to the manufacturer´s instructions. Purity was determined by flow cytometry. Following isolation, cells were stained with Cell Tracker Deep Red at a final concentration of 1 µM in 2 ml PEB. Cells were incubated for 20 min at 37 °C in darkness with shaking every 5 min. To remove excess dye, cells were washed three times using 5 ml PEB and finally resuspended in 400 µl PBS. Cells were counted with a ProCyte IDEXX Haematology Analyzer and 200 µl containing 4–5 × 10^6^ labeled CD4^+^ T cells were injected intravenously into the recipient mouse 4 h prior to imaging time.

Shortly before the desired imaging time, the recipient mouse was anesthetized by a i.p. injection of Ketamine and Xylazine. Hair on the back of the hindlegs was removed and the animal was transferred to an imaging stage in the prone position. After a skin incision above the knee, connective and fat tissue were moved apart to expose the popliteal LN below. Using sharp forceps, a small hole was created beneath the LN and filled with wet tissue pieces to lift the LN out of the surrounding tissue. The area was kept moist by draping wet tissues around the skin incision. A cover glass (Corning) was placed on top of the exposed LN and fixed in the imaging stage. Multi-photon microscopy was performed at the core facility bioimaging of the Biomedical Center of the LMU Munich with a Leica SP8 MP microscope (Leica Application Suite), equipped with a pulsed InSight DS + laser. Excitation of Cell Tracker Deep Red was performed at 1050 nm. Emission was filtered through a main beam splitter at 560 nm, followed by a 488 LPXR beamsplitter or RSP 620 filter. The bandpass filters used for detection were FITC: 525/50, CellTracker Deep Red: 650/50. Images were acquired using a 20x objective with a 1.0 NA, image pixel size was 200 nm and images were recorded with external, non-descanned hybrid photo detectors (HyDs). As a reference, the Second Harmonic Generation signal of the LN capsule was used. 104 µm z-stacks were recorded with a step size of 4 µm, for a duration of 10 min.

### Proliferation assays

96-well plates were coated with 2 µg/ml anti-CD3 antibody in PBS (or PBS alone as control) and incubated overnight at 4 °C. Mouse spleen and LNs (pooled from inguinal, axillary, and brachial LNs) were harvested at the indicated time points and passed through a 40 µm cell strainer to obtain single-cell suspensions. Purified T cell populations (CD4^+^ or CD8^+^) were generated using EasySep^TM^ Mouse T Cell Isolation kits according to the manufacturer’s instructions. The unbound fraction of each isolation was adjusted to 2 × 10^6^ cells/ml in PBS and stained with an equal volume of Cell Trace Violet at a final concentration of 5 µM. Cells were stained for 15 min at 37 °C in darkness with shaking every 5 min. Following staining, 10 ml of complete medium was added and cells incubated for a further 5 min to absorb any unbound dye. Cells were then centrifuged for 5 min at 300 × *g*, washed with complete medium (RPMI, 10% FCS, 2 mM L-glutamine, 1% penicillin & streptomycin, 50 µM β-mercaptoethanol), and resuspended at 5 × 10^5^ cells/ml in complete medium. Plate wells were then washed with PBS to remove unbound anti-CD3, and 100 µl cell suspension added. Control wells were topped up with an additional 100 µl media, and stimulated wells were given 100 µl anti-CD28 antibody diluted to 4 µg/ml in media. Plates were incubated at 37 °C with 5% CO_2_ for 2.5 days to divide prior to analysis.

For flow cytometry analysis, cells were transferred to V-bottom plate and washed with PEB (PBS with 1% BSA and 2 mM EDTA). All antibodies were diluted in PEB. Non-specific binding was blocked with 10 µg/ml anti-CD16/32 for 10 min at room temperature. An equivalent volume of 2x staining mix was added, containing anti-mouse CD25-AlexaFluor488, anti-mouse CD4-APC, anti-mouse CD8-APC/Cy7, and anti-mouse-B220-PE/Cy7, and cells were incubated at 4 °C for 30 min. Wells were washed twice with PEB and cells were resuspended in 200 µl PEB + 100 µl Propidium Iodide viability dye (5 µg/ml)and analyzed on a Gallios 10-channel flow cytometer (Beckman Coulter).

### Protein quantification

Samples were collected at the indicated time points and immediately transferred into Milenyi gentleMACS M tubes (Miltenyi Biotec) filled with 500 μl of T-PER medium and supplemented with protease inhibitors. Samples were homogenized using the gentleMACS Octo Dissociator (Miltenyi Biotec) and immediately stored on ice. Proteins were quantified using the Pierce BCA Protein Assay Kit at 1:2 and 1:5 dilutions, according to the manufacturer’s instructions.

### Proteomics

Mouse LNs (pooled from inguinal, axillary, and brachial LNs) were harvested at the indicated time points and passed through a 40 µm cell strainer to obtain single-cell suspensions. Purified T cell populations (CD4^+^) were generated using the EasySep Mouse CD4^+^ T Cell Isolation kit according to the manufacturer’s instructions.

Samples were subjected to in-solution digestion and purified through C18 STAGE-tip (EmporeTM, IVA-Analysentechnik, Meerbusch, Germany)^27,28^. LC-MS/MS was performed on a Q Exactive HF quadrupole orbitrap mass spectrometer coupled to a nanoflow UHPLC instrument (Easy nLC; Thermo Fisher Scientific, Waltham, MA, USA). Eluted peptides were separated over a gradient for 3 h on a 50 cm C18 column.

The mass spectra obtained were processed using the MaxQuant computational platform (v. 1.5.6.2i)^29,30^ and Perseus analysis software (v. 1.5.5.5)^31^. Spectra were searched against the Mus musculus Uniprot sequence databases (Aug. 2017; 53,219 sequences) using the Andromeda search engine^32^. Quantification was performed by using the label-free quantification (LFQ) algorithm^33^ and match between runs was selected.

Protein quantities were log_2_ transformed and samples were filtered for replicate reproducibility. Rows were filtered based on valid values of 3 in at least one group, and an imputation step performed to replace missing values from a normal distribution. Statistical analysis was performed using an unpaired two-sided Student’s t-test to compare differentially expressed proteins between collection times, with *P* < 0.05 at a false discovery rate of 0.05 and background variance parameter S0 = 1. Student’s t-test differences were used in a GO 1D annotation enrichment with an FDR cut-off of 0.01 to assess global de-/enrichment towards biological processes (GOBP) categories. A principal component analysis was performed on log_2_ transformed LFQ intensities with an FDR cut-off of 0.05.

### Chromatin Immunoprecipitation

Samples were collected 6 h following FITC administration to the ear. Draining LNs were snap-frozen on dry ice and stored at −80 °C prior to processing. Tissue was Dounce homogenized in 1 ml of Homogenization buffer (10 mM Hepes-KOH, 10 mM KCl, 5 mM MgCl_2_, 0.5 mM DTT, 1x complete TM Protease Inhibitor). The homogenate was fixed in 1% formaldehyde in PBS. Nuclei were isolated and chromatin suspensions were obtained through sonication (Diagenode Bioruptor) to obtain fragments of 0.2–0.8 kb in size. Immunoprecipitation was performed with anti-mouse/human BMAL1 antibody or control IgG. DNA concentration was determined by using a QUBIT dsDNA HS kit (Thermo Fisher Scientific). Real-time ChIP–quantitative PCR was performed by use of SYBR Green Master Mix (Roche) in LightCycler 480 II (Roche). Occupancy of BMAL1 at the *Icam1* promoter region was quantified by qPCR targeting regions identified as containing E-boxes using the SCOPE motif finder (sequences are provided in Supplementary Table 2). Relative enrichment was determined as the fold change between BMAL1 and control antibody samples.

### Vaccination

Commercial hepatitis A vaccine (HAVRIX, GlaxoSmithKline) was obtained via the clinic at the University Hospitals of Geneva (HUG). Mice were anesthetized with isoflurane inhalation and 50 µl of the proprietary vaccine formula was injected into the thigh muscle. As an adjuvant control, 50 µl of Alhydrogel adjuvant 2% diluted 1:20 in PBS was given to provide the equivalent alum content.

A SARS-CoV-2 vaccine was formulated in house using recombinant spike receptor binding domain (RBD) protein (aa319–591, kindly provided by the protein production core facility of the Ecole Polytechnique Fédérale de Lausanne (EPFL)) adsorbed to Alhydrogel adjuvant 2% to generate an emulsion with a concentration of 200 µg/ml. Mice were anesthetized with isoflurane inhalation and 50 µl of the vaccine formulation was injected into the thigh muscle.

28 days following vaccination, mice were euthanised and blood was collected from the vena cava. Blood samples were allowed to clot for 30 min at room temperature, then centrifuged at 10,000 × *g* for 10 min. Serum supernatant was aliquoted and frozen at −80 °C prior to analysis.

### Splenocyte restimulation assay

Spleens were harvested on day 28 after vaccination. Samples were processed through 70 µm cell strainers (Corning) with an excess volume of PEB (PBS with 1% BSA and 2 mM EDTA) and centrifuged at 300 × *g* for 5 min. Cell pellets were resuspended in 5 ml RBC lysis buffer (155 mM NH_4_Cl, 0.1 mM KHCO_3_, 0.1 mM EDTA in ddH_2_O) and incubated for 5 min at room temperature. 5 ml PEB was then added to quench the lysis reaction and samples centrifuged again.

Splenocytes were plated at 1 × 10^6^ cells/well in a round-bottom 96-well plate in RPMI (supplemented with 10% FCS, 1% Pen/Strep, 1 mM sodium pyruvate, 50 µM β-mercaptoethanol and 20 U/ml IL-2). Cells were cultured for 72 h with the vaccine antigen (HAV antigen or recombinant spike RBD protein (aa319–591)), 1 µg/ml in 200 µl total volume. GolgiPlug™ (1:1000) was added for the final 4 h of incubation.

Following stimulation, cells were washed and processed for flow cytometry staining. Cells were resuspended in fixable viability dye in PBS for 15 min at room temperature, followed by membrane staining for 15 min at 4 °C. Cells were washed and fixed/permeabilised for 15 min at room temperature (Foxp3/Transcription Factor Staining Buffer Set). Intracellular staining was performed for 45 min at room temperature. Cells were washed and resuspended in 300 µl PEB and analyzed using an 18-color BD LSR Fortessa (BD Biosciences) flow cytometer. Acquired data was analyzed using FACSDiva 8 (BD Biosciences) and FlowJo 10 (TreeStar) software.

### ELISA

An ELISA to detect mouse anti-HAV IgG was established in house using HAV antigen. Briefly, 96-well plates were coated with 5 µg/ml HAV antigen and incubated overnight at 4 °C. Wells were then washed with PBS-Tween 0.05% and incubated with blocking solution (PBS-Tween 0.05% with 1% BSA) for 1 h at 37 °C. Wells were then washed and 50 µl sample or standard added to the relevant wells. Samples were assayed individually at 8 concentrations (serial 1:2 dilutions in blocking solution starting from 1:100) to ensure a readout within the assay range. A pooled sample of sera from vaccinated mice served as standard and inter-plate calibrator. Following incubation for 90 min at 37 °C, wells were washed and 50 µl HRP-conjugated goat anti-mouse IgG detection antibody applied (1:1000 in blocking buffer). The plates were incubated for 1 h at 37 °C, washed, and detection substrate solution added (1 mg/ml ABTS in 30 mM citric acid, 44 mM Na_2_HPO_4_.12H_2_O, and 0.0001% H_2_O_2_). Following a 1 h incubation, the absorbance at 405 nm measured using a SpectraMax Plus plate reader and SoftMax Pro software (Molecular Devices).

### Mathematical modeling and parameter estimation

All mathematical models used to analyze the data and predict varying dynamics, as well as the parameter estimation methods, are explained in detail in the corresponding Supplementary Figs. 3–4, 7 and Supplementary Note 1. The mathematical model determining the rhythmic homing dynamics of T cells (ID1) was based on a previous model^7^. Homing of DC to LNs was determined using a mathematical model describing the dynamics observed within the crawl-In assays provided by Holtkamp et al.^18^ (ID2). Furthermore, measurements on the division index (Fig. 3c) were used to determine the oscillating dynamics of T cell proliferation (ID3). All individual components were combined within a final model explaining individual cell interactions and influence of rhythmicity on the dynamics (ID4). Models (ID1-ID3) were fitted to the data using a Maximum-Likelihood approach with parameter estimates obtained by profile likelihood analyses^34^. All individual parameters, especially those defining the phase-shift of the rhythmic components, were identifiable. Analyses were performed in R relying on the packages *deSolve* and *ProfileIroning* (https://github.com/GabelHub/ProfileIroning).

### Statistics and reproducibility

Statistical analyses were generally performed using GraphPad Prism v9 unless otherwise specified. Analyses were performed by two-way ANOVA, one-way ANOVA or Student’s t-test with *P < 0.05, **P < 0.01, ***P < 0.001, and ****P < 0.0001. Data are plotted as mean ± standard error of mean (SEM).

Exact *n* values and number of independent experiments performed for each experiment are provided below:

Figure 1: (a) Every group is composed of *n* = 3 mice from 5 independent experiments. (b) For lymph nodes collected: 12 h post-treatment, *n* = 3 mice for all ZTs; 24 h post-treatment, *n* = 5 mice for ZT1, ZT17, and ZT13 and *n* = 3 mice for ZT19; 48 h post-treatment, *n* = 5 mice for ZT1, *n* = 9 mice for ZT7 and ZT13 and *n* = 6 mice for ZT19; data collected from 5 independent experiments. (c) For lymph nodes collected after injection of: 10^2^ and 10^3^ BMDCs, *n* = 3 mice for both ZTs; 10^4^ BMDCs, *n* = 2 mice for both ZTs; 10^5^ BMDCs, *n* = 2 mice for ZT7 and *n* = 3 mice for ZT19. (d) For lymph nodes collected: 12h post-injection, *n* = 3 mice for both ZTs; 24 h post-injection, *n* = 10 mice for ZT7 and *n* = 8 mice for ZT19; 48 h post-injection, *n* = 14 mice for ZT7 and *n* = 10 mice for ZT19; data collected from 5 independent experiments. (e) Every group is composed of *n* = 8 mice. (f) For unstimulated lymph nodes, *n* = 736 tracks at ZT7 and *n* = 777 tracks at ZT19; while for FITC-stimulated lymph nodes, *n* = 512 tracks at ZT7 and *n* = 186 tracks at ZT19. (g) For unstimulated lymph nodes, *n* = 698 tracks at ZT7 and *n* = 689 tracks at ZT19; while for FITC-stimulated lymph nodes, *n* = 436 tracks at ZT7 and *n* = 173 tracks at ZT19. (j) Every group is composed of *n* = 9 mice from 3 independent experiments.

Figure 2: (a) For lymph nodes collected at ZT7 from mice that did not receive integrin-blocking treatment (control), *n* = 4 mice; while for all the other lymph nodes collected, *n* = 5 mice. (b) For lymph nodes collected from mice that did not receive integrin-blocking treatment (control), *n* = 3 mice at ZT7 and *n* = 2 mice at ZT19; for mice that received the integrin-blocking treatment *n* = 3 mice for both ZTs. (c) Every group is composed of *n* = 3 mice. (d) Every group is composed of *n* = 3 mice. (e) Every group is composed of *n* = 5 mice. (f) For lymph nodes collected from WT mice, *n* = 3 mice for ZT7, and *n* = 4 mice for ZT19; for lymph nodes collected from endothelial cell-specific *Bmal1*^*−/−*^ mice (BMAL1^ΔEC^), *n* = 3 mice for both ZTs. (g) Every group is composed of *n* = 3 mice. (h) Every group is composed of *n* = 3 mice; data collected from 2 independent experiments. (i) Every group is composed of *n* = 5 mice. (j) For lymph nodes harvested from mice that received the isotype control antibody, *n* = 13 mice for ZT7, and *n* = 8 mice for ZT19; for lymph nodes harvested from mice that received the anti-TNF antibody, *n* = 10 mice for ZT7, and *n* = 9 mice for ZT19; data collected from 2 independent experiments.

Figure 3: (a) Every group is composed of *n* = 5 mice. (b) Every group is composed of *n* = 5 mice. (c) For lymph nodes collected at ZT1, *n* = 3 mice; at ZT7, *n* = 6 mice; at ZT13, *n* = 6 mice; and at ZT19, *n* = 3 mice; data collected from 2 independent experiments. (d) For lymph nodes collected from WT animals, *n* = 4 mice for both ZTs; for lymph nodes collected from T cell-specific *Bmal1*^*−/−*^ mice (BMAL1^ΔTcell^), *n* = 3 mice for both ZTs.

Figure 4: (c) Every group is composed of *n* = 3 mice. (e) For lymph nodes collected from WT animals, *n* = 6 mice for ZT7, and *n* = 7 mice for ZT19; for lymph nodes collected from T cell-specific *Bmal1*^*−/−*^ mice (BMAL1^ΔTcell^), *n* = 3 mice for both ZTs; data collected from 3 independent experiments. (f) For lymph nodes collected from WT animals, *n* = 7 mice for ZT7, and *n* = 11 mice for ZT19; for lymph nodes collected from T cell-specific *Bmal1*^*−/−*^ mice (BMAL1^ΔTcell^), *n* = 5 mice for ZT7, and *n* = 4 mice for ZT19; data collected from 2 independent experiments.

Figure 5: (a) For lymph nodes collected from WT animals, *n* = 6 mice for both ZTs; for lymph nodes collected from T cell-specific *Bmal1*^*−/−*^ mice (BMAL1^ΔTcell^), *n* = 5 mice for ZT7, and *n* = 6 mice for ZT19; data collected from 2 independent experiments. (b) For WT mice vaccinated with adjuvants only, *n* = 6 mice for both ZTs; for WT mice vaccinated with the commercial HAVRIX vaccine, *n* = 22 mice for ZT7, and *n* = 20 mice for ZT19; for T cell-specific *Bmal1*^*−/−*^ mice (BMAL1^ΔTcell^) vaccinated with the commercial HAVRIX vaccine, *n* = 4 mice for both ZTs; for WT mice vaccinated with the commercial HAVRIX vaccine and anti-integrin blocking antibodies, *n* = 5 mice for ZT7, and *n* = 4 mice for ZT19; data collected from 4 independent experiments. (c) For WT mice vaccinated with the commercial HAVRIX vaccine, *n* = 5 mice for ZT7, and *n* = 7 mice for ZT19; for T cell-specific *Bmal1*^*−/−*^ mice (BMAL1^ΔTcell^) vaccinated with the commercial HAVRIX vaccine, *n* = 4 mice for both ZTs; for WT mice vaccinated with the commercial HAVRIX vaccine and anti-integrin blocking antibodies, *n* = 5 mice for both ZTs; data collected from 2 independent experiments.

### Reporting summary

Further information on research design is available in the Nature Portfolio Reporting Summary linked to this article.

## Supplementary information

Supplementary Info

Reporting Summary

## Data Availability

All data generated in this study have been deposited in the Yareta database under accession code 10.26037/yareta:hs6p7nzpkfhrlavgdopkqhafpm. The proteomics data have been deposited to the ProteomeXchange consortium via the PRIDE partner repository with the dataset identifier PXD039050. Source data are provided with this paper.

## Source data

Source Data

## Code availability

All mathematical models used to analyze the data and predict varying dynamics, as well as the parameter estimation methods, are explained in detail in Supplementary material (Supplementary Figs. 3–4, 7 and Supplementary Note 1). Analyses were performed in R relying on the packages *deSolve* and *ProfileIroning* (https://github.com/GabelHub/ProfileIroning).

## References

[1] Man K, Loudon A, Chawla A (2016). Immunity around the clock. Science.

[2] Baxter M, Ray DW (2020). Circadian rhythms in innate immunity and stress responses. Immunology.

[3] Palomino-Segura M, Hidalgo A (2021). Circadian immune circuits. J. Exp. Med..

[4] Scheiermann C, Gibbs J, Ince L, Loudon A (2018). Clocking in to immunity. Nat. Rev. Immunol..

[5] Scheiermann C, Kunisaki Y, Frenette PS (2013). Circadian control of the immune system. Nat. Rev. Immunol..

[6] Hopwood TW (2018). The circadian regulator BMAL1 programmes responses to parasitic worm infection via a dendritic cell clock. Sci. Rep..

[7] Druzd D (2017). Lymphocyte circadian clocks control lymph node trafficking and adaptive immune responses. Immunity.

[8] Sutton CE (2017). Loss of the molecular clock in myeloid cells exacerbates T cell-mediated CNS autoimmune disease. Nat. Commun..

[9] Nobis CC (2019). The circadian clock of CD8 T cells modulates their early response to vaccination and the rhythmicity of related signaling pathways. Proc. Natl Acad. Sci. USA.

[10] de Bree LCJ (2020). Circadian rhythm influences induction of trained immunity by BCG vaccination. J. Clin. Invest..

[11] Lange T, Dimitrov S, Bollinger T, Diekelmann S, Born J (2011). Sleep after vaccination boosts immunological memory. J. Immunol..

[12] Lange T, Perras B, Fehm HL, Born J (2003). Sleep enhances the human antibody response to hepatitis A vaccination. Psychosom. Med..

[13] Long JE (2016). Morning vaccination enhances antibody response over afternoon vaccination: a cluster-randomised trial. Vaccine.

[14] Suzuki K, Hayano Y, Nakai A, Furuta F, Noda M (2016). Adrenergic control of the adaptive immune response by diurnal lymphocyte recirculation through lymph nodes. J. Exp. Med..

[15] MartIn-Fontecha A (2003). Regulation of dendritic cell migration to the draining lymph node: impact on T lymphocyte traffic and priming. J. Exp. Med..

[16] Ohl L (2004). CCR7 governs skin dendritic cell migration under inflammatory and steady-state conditions. Immunity.

[17] Ulvmar MH (2014). The atypical chemokine receptor CCRL1 shapes functional CCL21 gradients in lymph nodes. Nat. Immunol..

[18] Holtkamp SJ (2021). Circadian clocks guide dendritic cells into skin lymphatics. Nat. Immunol..

[19] Xu H (1994). Leukocytosis and resistance to septic shock in intercellular adhesion molecule 1-deficient mice. J. Exp. Med..

[20] Fortier EE (2011). Circadian variation of the response of T cells to antigen. J. Immunol..

[21] Prather AA (2012). Sleep and antibody response to hepatitis B vaccination. Sleep.

[22] Ruben MD, Smith DF, FitzGerald GA, Hogenesch JB (2019). Dosing time matters. Science.

[23] Benedict C, Cedernaes J (2021). Could a good night’s sleep improve COVID-19 vaccine efficacy?. Lancet Respir. Med..

[24] He W (2018). Circadian expression of migratory factors establishes lineage-specific signatures that guide the homing of leukocyte subsets to tissues. Immunity.

[25] Wyse C, O’Malley G, Coogan AN, McConkey S, Smith DJ (2021). Seasonal and daytime variation in multiple immune parameters in humans: Evidence from 329,261 participants of the UK Biobank cohort. iScience.

[26] Lutz MB (1999). An advanced culture method for generating large quantities of highly pure dendritic cells from mouse bone marrow. J. Immunol. Methods.

[27] Geddes-McAlister J, Gadjeva M (2019). Mass spectrometry-based quantitative proteomics of murine-derived polymorphonuclear neutrophils. Curr. Protoc. Immunol..

[28] Rappsilber J, Ishihama Y, Mann M (2003). Stop and go extraction tips for matrix-assisted laser desorption/ionization, nanoelectrospray, and LC/MS sample pretreatment in proteomics. Anal. Chem..

[29] Cox J, Mann M (2008). MaxQuant enables high peptide identification rates, individualized p.p.b.-range mass accuracies and proteome-wide protein quantification. Nat. Biotechnol..

[30] Tyanova S, Temu T, Cox J (2016). The MaxQuant computational platform for mass spectrometry-based shotgun proteomics. Nat. Protoc..

[31] Tyanova S (2016). The Perseus computational platform for comprehensive analysis of (prote)omics data. Nat. Methods.

[32] Cox J (2011). Andromeda: a peptide search engine integrated into the MaxQuant environment. J. Proteome Res..

[33] Cox J (2014). Accurate proteome-wide label-free quantification by delayed normalization and maximal peptide ratio extraction, termed MaxLFQ. Mol. Cell Proteom..

[34] Raue A (2009). Structural and practical identifiability analysis of partially observed dynamical models by exploiting the profile likelihood. Bioinformatics.

