# Supplementary material for: Influence of circadian clocks on adaptive immunity and vaccination responses
Source: Nat Commun. 2023 Jan 30;14:476. doi: 10.1038/s41467-023-35979-2 (PMC9885059; doi:10.1038/s41467-023-35979-2)
Supplement: Supplementary file 1 — Supplementary Info [file 41467_2023_35979_MOESM1_ESM.pdf]

Supplementary Information for

Influence of circadian clocks on adaptive immunity and vaccination responses

Louise Madeleine Ince[1,13,#], Coline Barnoud[1,#], Lydia Kay Lutes[1], Robert Pick[1], Chen Wang[1], Flore Sinturel[2,3,4,5], Chien-Sin Chen[6], Alba de Juan[6], Jasmin Weber[6], Stephan J. Holtkamp[6], Sophia Martina Hergenhan[6], Jennifer Geddes-McAlister[7,14], Stefan Ebner[7,8], Paola Fontannaz[1,9], Benjamin Meyer[1,9], Maria Vono[1,9], Stéphane Jemelin[1], Charna Dibner[2,3,4,5], Claire-Anne Siegrist[1,9], Felix Meissner[7,8], Frederik Graw[10,11], and Christoph Scheiermann[1,5,6,12] *.

[1] Department of Pathology and Immunology, Faculty of Medicine, University of Geneva, Geneva, Switzerland

[2] Department of Medicine, Division of Endocrinology, Diabetes, Nutrition and Patient Education, Faculty of Medicine, University of Geneva, Geneva, Switzerland

[3] Department of Cell Physiology and Metabolism, Faculty of Medicine, University of Geneva, Geneva, Switzerland

[4] Diabetes Center, Faculty of Medicine, University of Geneva, Geneva, Switzerland

[5] Institute of Genetics and Genomics of Geneva (iGE3), University of Geneva, Geneva, Switzerland

[6] Walter-Brendel-Centre of Experimental Medicine, Ludwig-Maximilians-University Munich, BioMedical Centre, Planegg-Martinsried, Germany

[7] Experimental Systems Immunology, Max Planck Institute of Biochemistry, Martinsried, Germany

[8] Systems Immunology and Proteomics, Institute of Innate Immunity, Medical Faculty, University of Bonn, Bonn, Germany

[9] World Health Organization Collaborating Center for Vaccine Immunology, Faculty of Medicine, University of Geneva, Geneva, Switzerland

[10] BioQuant - Center for Quantitative Biology, Heidelberg University, Heidelberg, Germany

[11] Interdisciplinary Center for Scientific Computing, Heidelberg University, Heidelberg, Germany

[12] Geneva Centre for Inflammation Research, Faculty of Medicine, University of Geneva, Geneva, Switzerland

[13] current address: Division of Pharmacology & Toxicology, College of Pharmacy, University of Texas at Austin, Austin, Texas, USA

[14] current address: Department of Molecular and Cellular Biology, University of Guelph, Guelph, Ontario, Canada

[#] These authors contributed equally to this work.

*** Contact:**

Christoph Scheiermann, PhD

University of Geneva

Centre Médical Universitaire (CMU)

Department of Pathology and Immunology (PATIM)

1, rue Michel-Servet

1206 Geneva, Switzerland

Tel: +41-2237-95747

Fax: +41-22-37-95746

christoph.scheiermann@unige.ch or

christoph.scheiermann@med.uni-muenchen.de

This Supplementary Information document contains:

- **Supplementary Figures S1-8**
- **Supplementary Tables S1-4**
- **Supplementary Note 1**

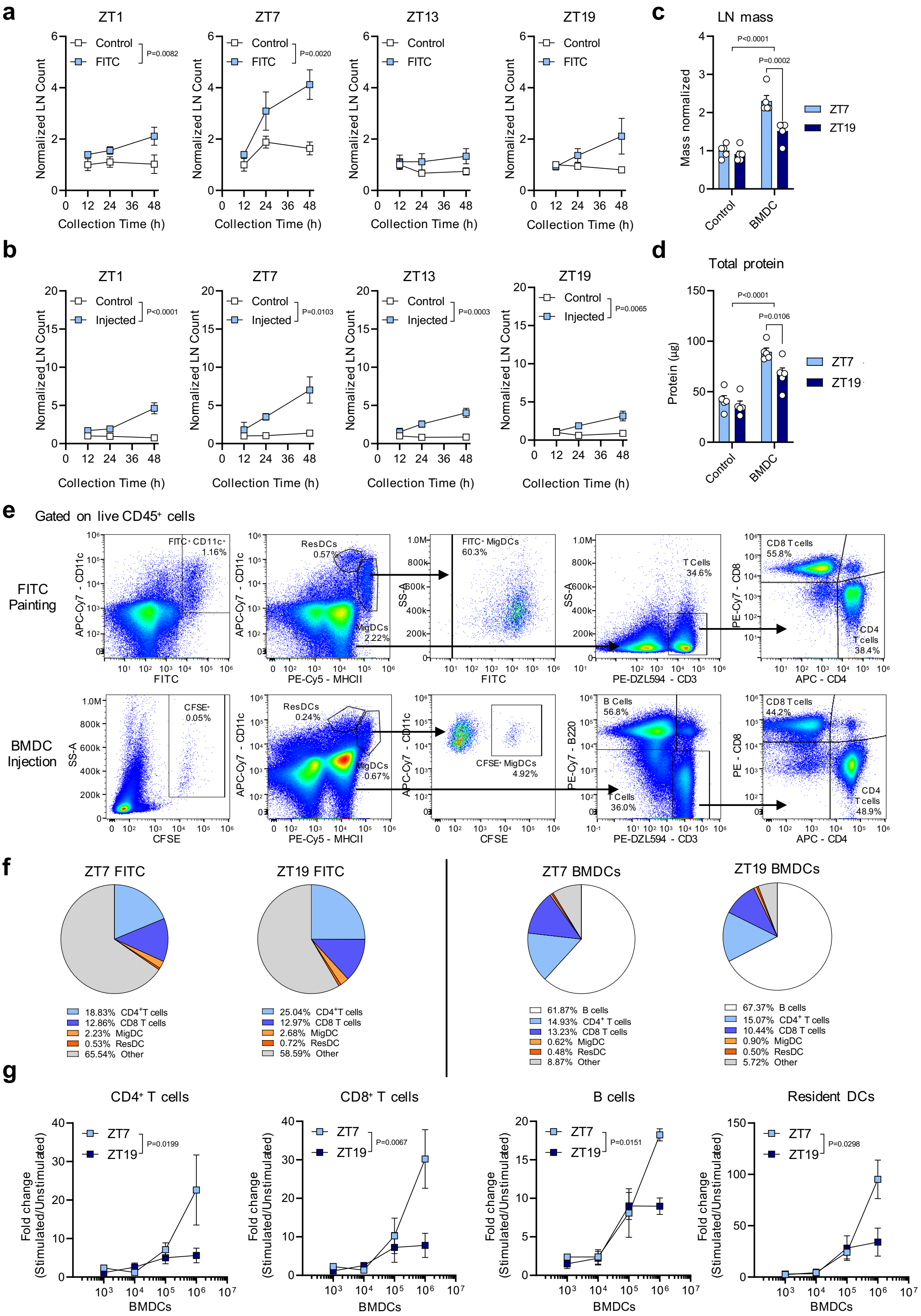
a
ZT1
ZT7
ZT13
ZT19
Control
FITC
P=0.0082
P=0.0020
Normalized LN Count
Collection Time (h)
c
LN mass
P<0.0001
P=0.0002
Mass normalized
Control
BMDC
ZT7
ZT19
b
Control
Injected
P<0.0001
P=0.0103
P=0.0003
P=0.0065
d
Total protein
P<0.0001
P=0.0106
Protein (µg)
e
Gated on live CD45⁺ cells
FITC Painting
BMDC Injection
FITC⁺ CD11c⁺ 1.16%
ResDCs 0.57%
MigDCs 2.22%
FITC⁺ MigDCs 60.3%
T Cells 34.6%
CD8 T cells 55.8%
CD4 T cells 38.4%
CFSE⁺ 0.05%
ResDCs 0.24%
MigDCs 0.67%
CFSE⁺ MigDCs 4.92%
B Cells 56.8%
T Cells 36.0%
CD8 T cells 44.2%
CD4 T cells 48.9%
APC-Cy7 - CD11c
FITC
PE-Cy5 - MHCII
SS-A
PE-DZL594 - CD3
PE-Cy7 - CD8
APC - CD4
CFSE
PE-Cy7 - B220
PE - CD8
f
ZT7 FITC
18.83% CD4⁺T cells
12.86% CD8 T cells
2.23% MigDC
0.53% ResDC
65.54% Other
ZT19 FITC
25.04% CD4⁺T cells
12.97% CD8 T cells
2.68% MigDC
0.72% ResDC
58.59% Other
ZT7 BMDCs
61.87% B cells
14.93% CD4⁺ T cells
13.23% CD8 T cells
0.62% MigDC
0.48% ResDC
8.87% Other
ZT19 BMDCs
67.37% B cells
15.07% CD4⁺ T cells
10.44% CD8 T cells
0.90% MigDC
0.50% ResDC
5.72% Other
g
CD4⁺ T cells
CD8⁺ T cells
B cells
Resident DCs
P=0.0199
P=0.0067
P=0.0151
P=0.0298
Fold change (Stimulated/Unstimulated)
BMDCs

Supplementary Figure 1. DC migration during the day elicits greater lymph node expansion

a, Time course of cell counts in parotid lymph nodes (LNs) following topical application of FITC, normalized to the 12 h time point on the contralateral side; n=3 mice, two-way ANOVA with Sidak's post test. **b,** Time course of cell counts in popliteal LNs following subcutaneous injection of 1x10^6 bone marrow-derived dendritic cells (BMDC), normalized to the 12 h time point on the contralateral side. For lymph nodes collected: 12h post-treatment, n=3 mice for all ZTs; 24h post-treatment, n=5 mice for ZT1, ZT17 and ZT13 and n=3 mice for ZT19; 48h post-treatment, n=5 mice for ZT1, n=9 mice for ZT7 and ZT13 and n=6 mice for ZT19; data collected from 5 independent experiments; two-way ANOVA with Sidak's post test. **c,** Popliteal LN mass 24 h following subcutaneous injection of 1x10^6 BMDCs, normalized to average control ZT7 mass; n=5 mice, two-way ANOVA with Sidak's post test. **d,** Total protein quantification in popliteal LN 24 h after subcutaneous injection of 1x10^6 BMDC; n=5 mice, two-way ANOVA with Sidak's post test. **e,** Gating strategies for flow cytometry analyses of lymph nodes following topical FITC application (top) or subcutaneous CFSE-labelled BMDCs (bottom). **f,** Frequencies of cell populations within lymph nodes 48 h following topical FITC application (left) or subcutaneous injection of CFSE-labelled BMDCs (right). **g,** Dose-response curve of cell subsets in the LN 48 h after subcutaneous injection of BMDCs, normalized to the contra-lateral side. For lymph nodes collected after injection of: 10^2 and 10^3 BMDCs, n=3 mice for both ZTs; 10^4 BMDCs, n=2 mice for both ZTs; 10^5 BMDCs, n=2 mice for ZT7 and n=3 mice for ZT19; two-way ANOVA with Sidak's post test. Data are plotted as mean ± standard error of mean (SEM); ns, not significant. Source data are provided as a Source Data file.

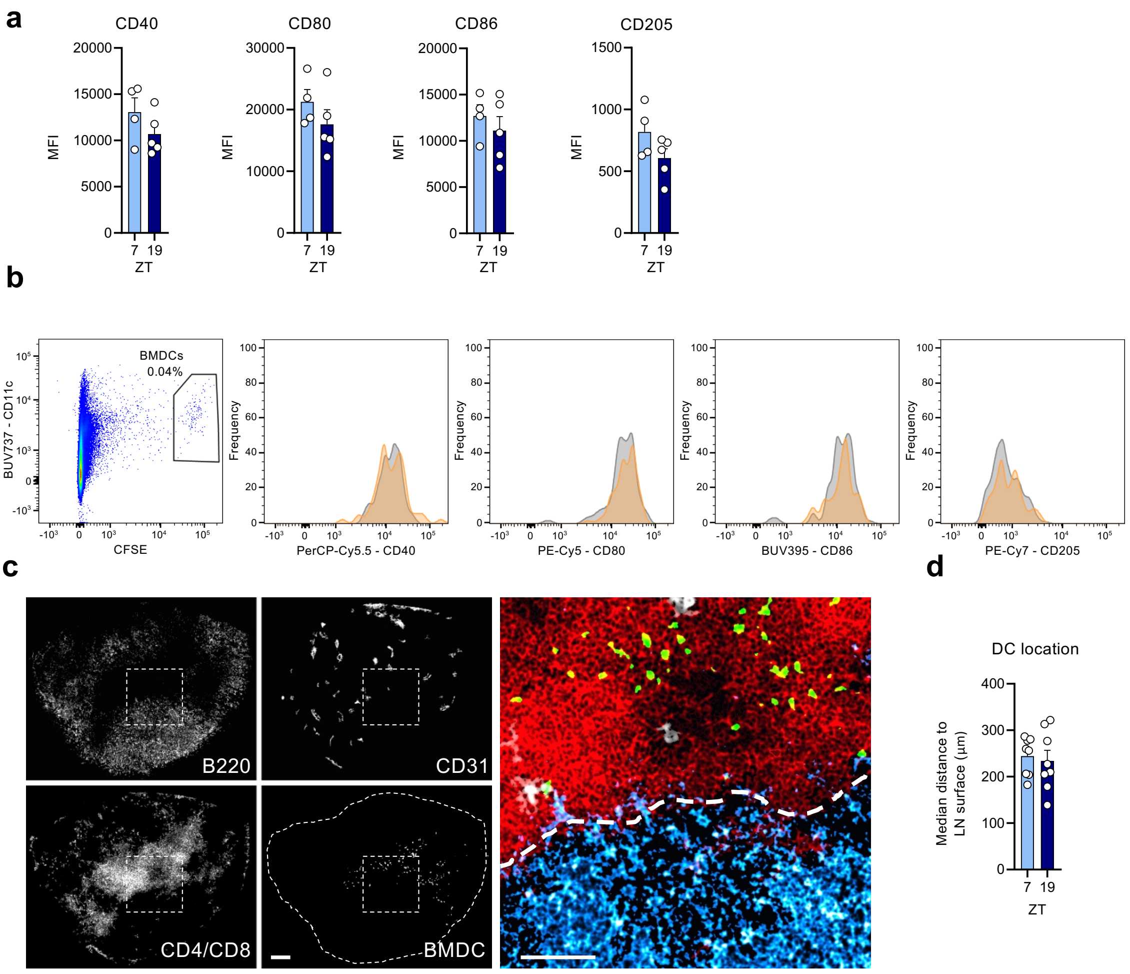
a
CD40
CD80
CD86
CD205
MFI
ZT
7
19
b
BMDCs
0.04%
BUV737 - CD11c
CFSE
Frequency
PerCP-Cy5.5 - CD40
PE-Cy5 - CD80
BUV395 - CD86
PE-Cy7 - CD205
c
B220
CD31
CD4/CD8
BMDC
d
DC location
Median distance to LN surface (µm)

Supplementary Figure 2. Phenotype and location of injected BMDCs

a, Phenotype of migrated cells 24 h after subcutaneous injection of $1x10^6$ CFSE-labelled bone marrow-derived dendritic cells (BMDCs). For lymph nodes harvested at ZT7, n=4 mice, and for lymph nodes harvested at ZT19, n=5 mice; unpaired two-sided Student's t-test. **b**, Gating strategy for phenotypic analysis of migrated ($CFSE^+$) cells shown in panel a. **c**, Representative images of popliteal lymph nodes collected 24 h following subcutaneous injection of $1x10^6$ CFSE-labelled BMDCs (green), counterstained with antibodies against mouse CD31 (PECAM, white), CD4 and CD8 (red), and B220 (blue). Scale bars = 50 µm. **d**, Median distribution of DC distance from capsule of the popliteal lymph node 24 h after subcutaneous injection of $1x10^6$ BMDCs; n=4 mice with 2 sections/mouse quantified; unpaired two-sided Student's t-test. Data are plotted as mean ± standard error of mean (SEM); ns, not significant. Source data are provided as a Source Data file.

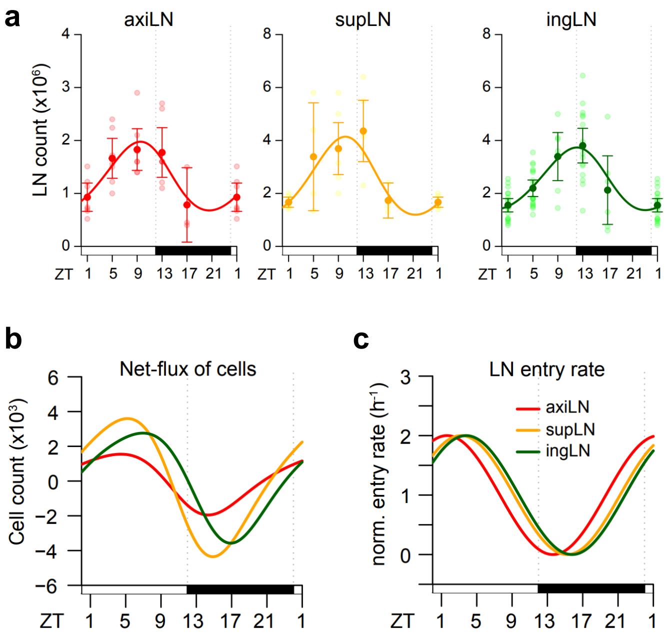

Rhythmic homing dynamics of T cells (ID1)

The mathematical model used to describe the homing and egress dynamics of T cells for skin-specific lymph nodes is based on the model described in Druzd el al.[7]. In this previous study, it was found that homing and egress of lymphocytes are both assumed to follow sinusoidal oscillating dynamics. The lymphocyte count within an individual lymph node, *X(t)*, therefore changes over time according to

$$\frac{dX}{dt} = A\left(1+\sin(\omega(t+\phi_h))\right) - d\left(1+\sin(\omega(t+\phi_e))\right)X \qquad (1)$$

Hereby, *A* and *d* define the homing and egress rates of cells, respectively, and ω the angular frequency of the oscillating dynamics given a 24h-cycle. The individual phases of the time-dependent gain and loss rates of lymphocytes are shifted by the parameters ϕ_h and ϕ_e, respectively.

The model in Eq. (1) was fitted to the experimental data on the lymphocyte counts of the axillary, inguinal and superficial cervical lymph node[7] using the `deSolve` and `optim`-package in R. Parameter estimates for the individual lymph nodes are given in **Table S1**, with 95%-confidence intervals of estimates obtained by profile likelihood analysis[33]. Individual data and best model predictions are shown.

Supplementary Figure 3. Rhythmic homing dynamics of T cells

a, Determination of rhythmic T cell homing and egress dynamics for axillary (axi, red), superficial cervical (sup, orange), and inguinal (ing, green) lymph nodes (LNs) using the mathematical model given in Eq. (1) (ID1). Individual measurements (light dots), mean ± 1.96×SEM (solid dots, arrows), and best model prediction (solid line) are shown. Data are from Druzd et al.[7]. For axillary lymph nodes, n=7 mice for ZT1, ZT5, ZT9 and ZT13; and n=3 mice for ZT17. For superior cervical lymph node, n=6 mice for ZT1 and ZT13; n=4 mice for ZT5; n=7 mice for ZT9 and n=3 mice for ZT17. For inguinal lymph nodes, n=18 mice for ZT1; n=19 mice for ZT5; n=7 mice for ZT9; n=16 mice for ZT13 and n=6 mice for ZT17. Data were collected from 10 independent experiments. **b**, Predicted net-flux of cells in and out of the LN combining rhythmic homing and egress. **c**, Normalized entry rate showing highest influx rate around ZT 2-7. Data are plotted as mean ± standard error of mean (SEM). Source data are provided as a Source Data file.

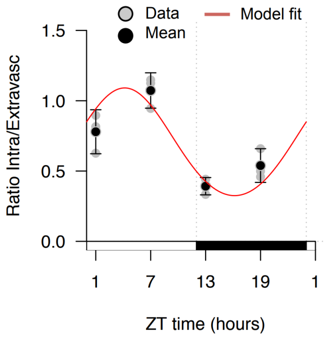

Homing dynamics of Dendritic cells (ID2)

The homing dynamics of dendritic cells (DC) was determined using the data of the crawl-In assay given in Holtkamp et al.[18]. In brief, labelled bone marrow-derived DC were injected into ears of mice that were harvested at different ZT times. The infiltration of DC into the vasculature was then followed by 2-photon microscopy. Data indicate a ZT-dependent infiltration of cells into the vasculature. Distinguishing between the extra- and intravascular concentration of DC, i.e., DC_E and DC_I, respectively, the dynamics after injection is then described by the following system of ordinary differential equations:

$$\frac{DC_E}{dt} = -\alpha\left(1 + \sin(\omega(t + \phi_{h,DC}))\right) DC_E - \mu DC_E \qquad (2)$$

$$\frac{DC_I}{dt} = \alpha\left(1 + \sin(\omega(t + \phi_{h,DC}))\right) DC_E + \mu DC_E \qquad (3)$$

Hereby, the parameter α defines the amplitude of the rhythmic component of the homing rate with angular frequency ω given a 24h-cycle and phase-shift parameter $\phi_{h,DC}$, while μ specifies a constant homing rate independent of the time of injection and ear harvest.

The model given by Eqs. (2) and (3) was fitted to the observed ratio of intra- and extravascular cells using the deSolve and optim-package in R. The initial concentration of DC injected was set to 1 without loss of generality, $DC_E(0) = 1$. Parameter estimates are given in **Table S1**, with 95%-confidence intervals of estimates obtained by profile likelihood analysis[33]. Individual data and best model predictions are shown.

Supplementary Figure 4. Homing dynamics of dendritic cells

Prediction of rhythmic dendritic cell (DC) influx to lymph nodes (LN) dependent on time of day (Zeitgeber time, ZT) plating of DCs based on ratio of intra- and extravascular DCs observed in the crawl-in assay data of publication [18]. Data were analyzed based on the mathematical model shown in Eq. (2) and (3). Individual measurements (n=3 mice per group; grey dots), mean ± 1.96 × SEM (solid dots, arrows), and best model prediction (red line) are shown. Parameter estimates are given in **Table S1**. Data are plotted as mean ± standard error of mean (SEM). Source data are provided as a Source Data file.

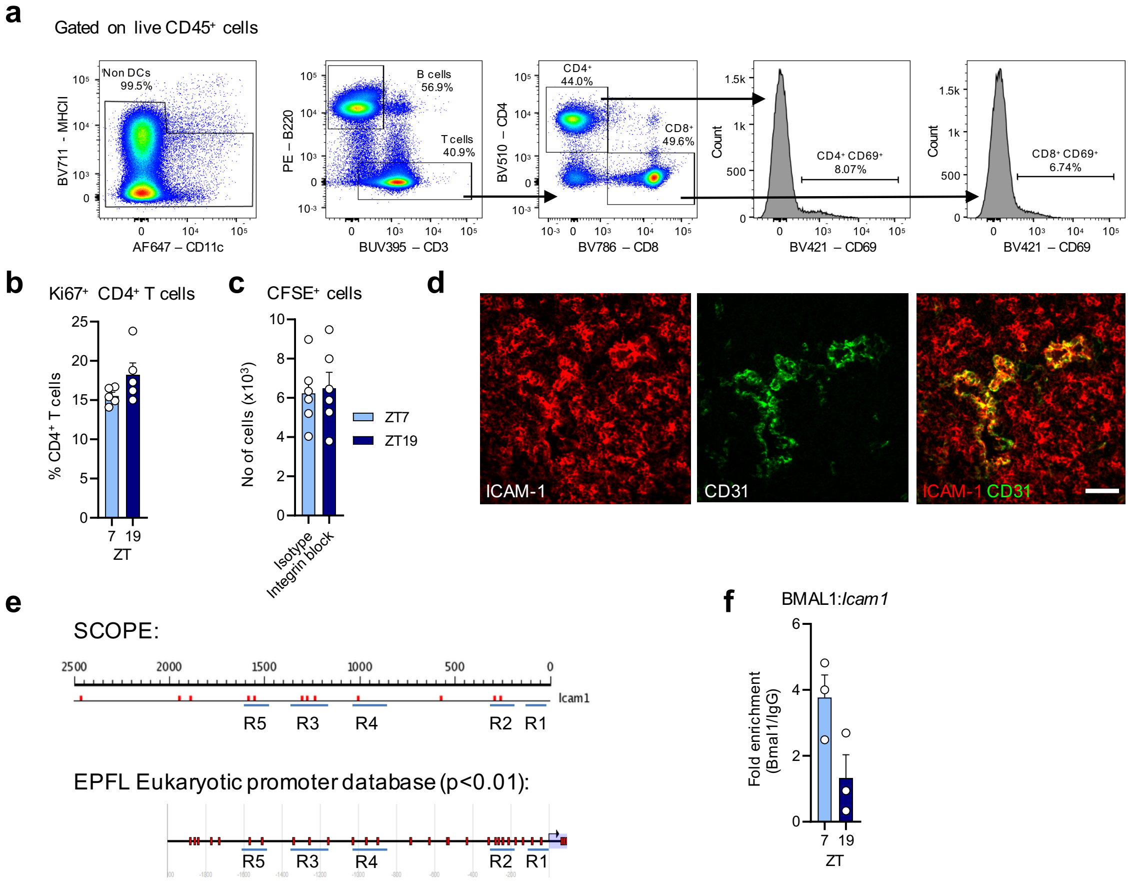
a
Gated on live CD45+ cells
Non DCs 99.5%
BV711 - MHCII
AF647 – CD11c
B cells 56.9%
T cells 40.9%
PE – B220
BUV395 – CD3
CD4+ 44.0%
CD8+ 49.6%
BV510 – CD4
BV786 – CD8
CD4+ CD69+ 8.07%
CD8+ CD69+ 6.74%
Count
BV421 – CD69
b
Ki67+ CD4+ T cells
% CD4+ T cells
7 19
ZT
c
CFSE+ cells
No of cells (x10³)
Isotype
Integrin block
ZT7
ZT19
d
ICAM-1
CD31
ICAM-1 CD31
e
SCOPE:
Icam1
R5 R3 R4 R2 R1
EPFL Eukaryotic promoter database (p<0.01):
R5 R3 R4 R2 R1
f
BMAL1:Icam1
Fold enrichment (Bmal1/IgG)
7 19
ZT

Supplementary Figure 5. Control of ICAM-1 expression in lymph nodes

a, Flow cytometry gating strategy for $CD69^+$ T cells of Fig. 1j. **b,** Percentage of $CD4^+$ T cells positive for Ki67 staining in the popliteal lymph nodes (LN) 24 h after subcutaneous injection of $1x10^6$ bone marrow-derived dendritic cells (BMDCs); n=5 mice, unpaired two-sided Student's t-test. **c,** Numbers of migrated ($CFSE^+$) BMDCs in the popliteal LN cellularity 24 h following subcutaneous injection of $1x10^6$ BMDCs at ZT7, with or without prior treatment with integrin blocking antibodies; n=6 mice, unpaired two-sided Student's t-test. **d,** Representative images of ICAM-1 for quantifications in $PECAM-1^+$ HEVs, scale bar = 50 µm. **e,** Identification of BMAL1 binding sites in the mouse *Icam1* promotor by SCOPE and EPFL eukaryotic promoter database. **f,** Chromatin immunoprecipitation (ChIP) analysis of BMAL1 binding to the *Icam1* promoter region R3 in the parotid LN 6 h after topical FITC application; n=3 mice, unpaired two-sided Student's t-test. Data are plotted as mean ± standard error of mean (SEM); ns, not significant. Source data are provided as a Source Data file.

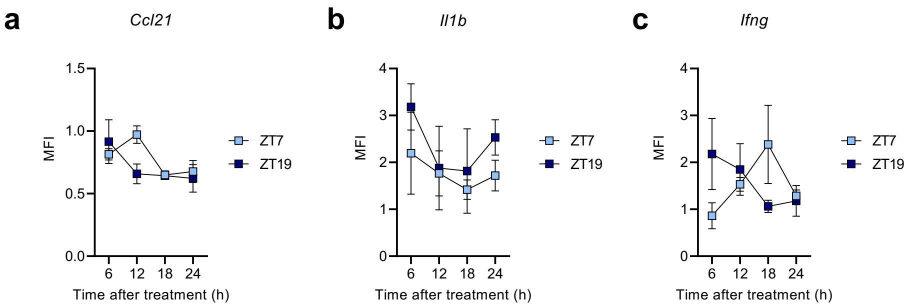
a
Ccl21
b
Il1b
c
Ifng
MFI
Time after treatment (h)
ZT7
ZT19
1.5
1.0
0.5
0.0
4
3
2
1
0
6
12
18
24

Supplementary Figure 6. Expression of pro-inflammatory genes

a-c, Time course of *Ccl21, Il1b and Ifng* mRNA expression in parotid lymph node after topical application of FITC; n = 3 mice, two-way ANOVA with Sidak's post test. Data are plotted as mean ± standard error of mean (SEM)); ns, not significant. Source data are provided as a Source Data file.

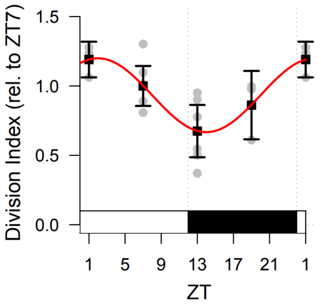

T cell proliferation dynamics (ID3)

To determine the observed rhythmic within the proliferation of T cells (**Figure 3c**, main manuscript), we describe the time-dependent oscillating dynamics of the division index, *DI*, relative to ZT7 by

$$\frac{DI}{dt} = DI_7 + \Lambda\left(1 + \sin(\omega(t + \phi_{h,P}))\right) \qquad (4)$$

Hereby, DI_7 defines the baseline division index at ZT7, and Λ, ω, and ϕ_P the amplitude, angular frequency and phase-shift of the rhythmic proliferation component, respectively. Parameters were estimated using a maximum likelihood approach as before in $\mathbb{R}$, with 95%-confidence intervals of estimates obtained by profile likelihood analysis[33]. Data and best model predictions are shown with parameter estimates given in **Table S1**.

Supplementary Figure 7. T cell proliferation dynamics

Rhythmic of T cell proliferation dynamics: T cell proliferation measured by the Division Index at different ZT time relative to the division observed at ZT7. Individual measurements (grey dots) and mean (±1.96 × SEM) of measurements are shown (black dots/arrows). Data collected at ZT1 and ZT19, n=3 mice per group; and for data collected at ZT7 and ZT13, n=6 mice per group. Data collected from 2 independent experiments. Red line indicates the best fit of a model assuming sinusoidal oscillating T cell proliferation dynamics (ID3) with parameter estimates given in **Table S1**. Data are plotted as mean ± standard error of mean (SEM). Source data are provided as a Source Data file.

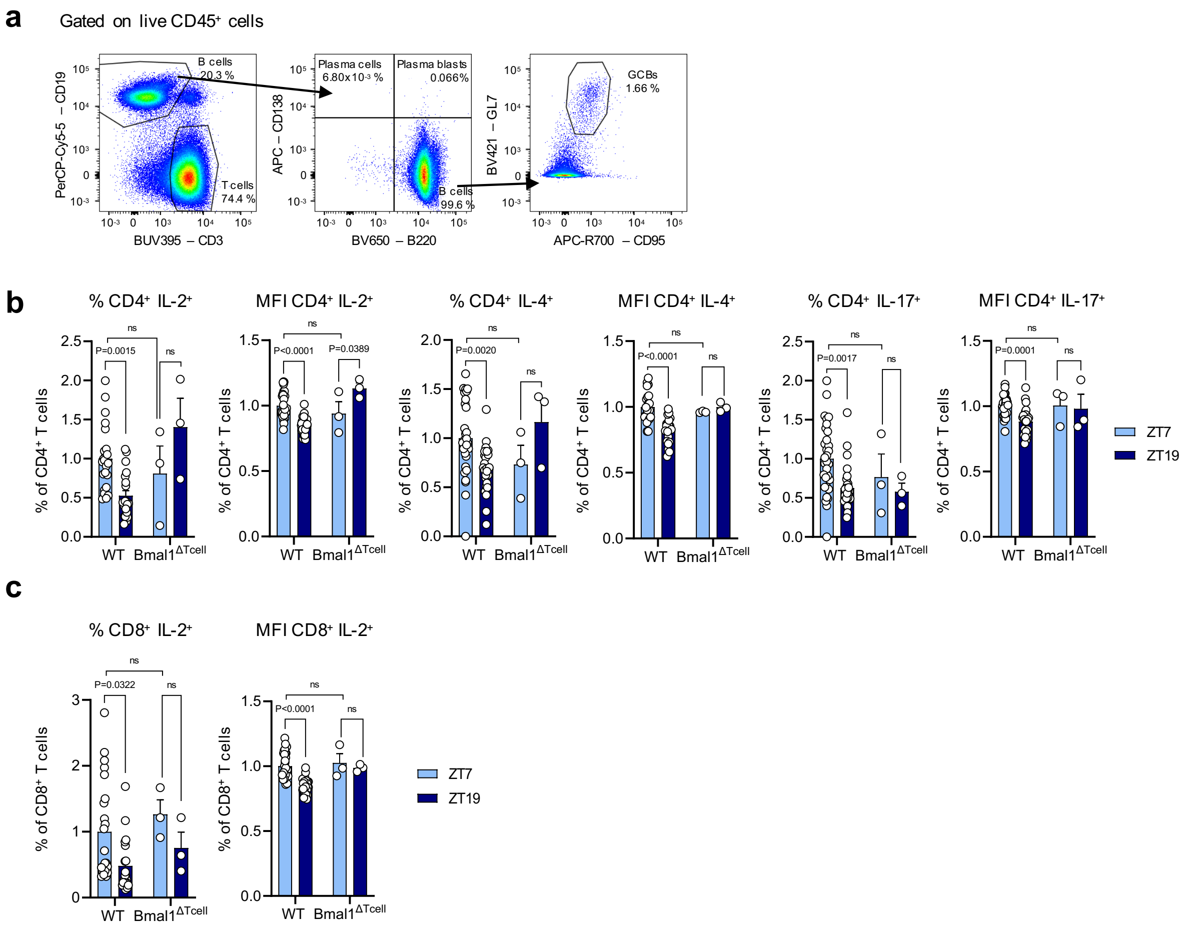
a
Gated on live CD45+ cells
B cells 20.3 %
T cells 74.4 %
PerCP-Cy5-5 – CD19
BUV395 – CD3
Plasma cells 6.80x10-3 %
Plasma blasts 0.066%
B cells 99.6 %
APC – CD138
BV650 – B220
GCBs 1.66 %
BV421 – GL7
APC-R700 – CD95
b
% CD4+ IL-2+
MFI CD4+ IL-2+
% CD4+ IL-4+
MFI CD4+ IL-4+
% CD4+ IL-17+
MFI CD4+ IL-17+
% of CD4+ T cells
P=0.0015
P<0.0001
P=0.0389
P=0.0020
P<0.0001
P=0.0017
P=0.0001
ns
WT
Bmal1ΔTcell
ZT7
ZT19
c
% CD8+ IL-2+
MFI CD8+ IL-2+
% of CD8+ T cells
P=0.0322
P<0.0001
ns
WT
Bmal1ΔTcell
ZT7
ZT19

Supplementary Figure 8. T cell responses after vaccination

a, Flow cytometry gating strategy for GCBs of Fig. 5a. **b,** $CD4^+$ T cell responses upon antigen restimulation 28 days after vaccination with an RBD protein-based SARS-CoV-2 vaccine. For T cells collected from WT mice, n=23, 31, 31, 23, 31, 31 mice for ZT7, and n=22, 28, 28, 20, 28, 28 mice for ZT19; for T cells collected from T cell-specific *Bmal1*$^{-/-}$ mice (BMAL1$^{\Delta Tcell}$) n=3 mice for both ZTs; data collected from 3 independent experiments;, two-way ANOVA with Tukey's post test. **c,** $CD8^+$ T cell responses upon antigen restimulation 28 days after vaccination with an RBD protein-based SARS-CoV-2 vaccine; For T cells collected from WT mice, n=23, 31 mice for ZT7, and n=20, 26 mice for ZT19; for T cells collected from T cell-specific *Bmal1*$^{-/-}$ mice (BMAL1$^{\Delta Tcell}$) n=3 mice for both ZTs; data collected from 3 independent experiments; one-way ANOVA with Tukey's post test. Data are plotted as mean ± standard error of mean (SEM); ns, not significant. Source data are provided as a Source Data file.

Supplementary Table 1. Parameter estimates for generated models

Numbers in brackets indicate 95% confidence interval of estimates.

**ID1: LN dynamics**			
	axiLN	supLN	ingLN
Homing, ϕ_h (in h)	4.4 [19.7, 6.9]	2.8 [20.8, 4.7]	2.2 [18.0,3.8]
Egress, ϕ_e (in h)	11.3 [8.4, 19.1]	11.3 [9.1, 17.2]	8.6 [6.3, 16.0]
**ID2: DC Influx**			
**Parameter**		Unit	Estimate
Homing, $\phi_{h,DC}$		h	1.34 [0.54, 2.20]
Rhythmic influx rate, α		$\times 10^{-1}$ h^{-1}	2.29 [2.13, 2.37]
Constant influx rate, μ		$\times 10^{-1}$ h^{-1}	2.81 [2.55, 3.07]
**ID3: Rhythmic cell proliferation**			
Phase transition, ϕ_P		h	4.0 [0.7, 8.4]
Amplitude, Λ of rhythmic proliferation rate			0.27 [0.06, 0.49]
Baseline Division Index, DI_0			0.93 [0.75, 1.11]
**ID4: Rhythmic cell interaction**			
Activation rate, σ		h^{-1}	0.011
Velocity factor ZT7, v_{ZT}		h^{-1}	1
Velocity factor ZT19, v_{ZT}		h^{-1}	0.75
Proliferation rate, ρ		h^{-1}	0.03
DC loss rate, γ		h^{-1}	0.02
Scaling factor for controlling efflux, λ			10^{-3}

Supplementary Table 2. Primer Sequences

Target	Primer 1 (F)	Primer 2 (R)	Primer 3 (R2)	Purpose
Bmal1 (flox)	ACTGGAAGTAACTTTATCAAACTG	CTGACCAACTTGCTAACAATTA		Genotyping (all transgenic lines)
Generic Cre	CGATGCAACGAGTGATGAGG	CGCATAACCAGTGAAACAGC		Genotyping (Cd4cre, Cd19cre, Lyz2cre, Cdh5-creERT2)
RosaEYFP	AAAGTCGCTCTGAGTTGTTAT	GGAGCGGGAGAAATGGATATG	AAGACCGCGAAGAGTTTGTC	Genotyping (RosaEYFP;Clec9cre)
Clec9cre	AAAAGTTCCACTTTCTGGATGATGA	TCACTTACTCCTCCATGCTGACG	GGCTCTCTCCCCAGCATCCACA	Genotyping (RosaEYFP;Clec9cre)
Rpl32	ACAATGTCAAGGAGCTGGAG	TTGGGATTGGTGACTCTGATG		qPCR
Icam1	GGACCACGGAGCCAATTTC	CTCGGAGACATTAGAGAACAATGC		qPCR
Tnfa	GCCTCTTCTCATTCCTGCTTG	CTGATGAGAGGGAGGCCATT		qPCR
Ccl21	TGAACAGACACAGCCCTCAAGA	CCTCTTTGCCTGTGAGTTGGA		qPCR
Il1b	TGTAATGAAAGACGGCACACC	TCTTCTTTGGGTATTGCTTGG		qPCR
Ifng	TCAAGTGGCATAGATGTGGAAGAA	TGGCTCTGCAGGATTTTCATG		qPCR
Icam1 R1	AATACCGAAGCCCTCGTTCC	GGGCGGCGCTTTTATAGTCT		BMAL1-Icam1 ChIP
Icam1 R2	ATCAGTTAACCAGGAGGCGTG	GGCCCCTGCGATCTAGGA		BMAL1-Icam1 ChIP
Icam1 R3	CAATCAAAACATCCCCGTGGAAA	GACGCCTATACAGTTGCTGTG		BMAL1-Icam1 ChIP
Icam1 R4	CTGCGAAAAGCAAGAGCAGT	TCTCACTCCTTTCCCCCACT		BMAL1-Icam1 ChIP
Icam1 R5	ATTCAGCTCTTGGACCGATTG	GTCACTGAGAGTCTTTGGTCCT		BMAL1-Icam1 ChIP

Supplementary Table 3. List of commercial reagents and kits used

Commercial reagents		
**Name**	**Company**	**Catalog #**
Acetone	Sigma-Aldrich	179124
Alhydrogel adjuvant	InvivoGen	vac-alu-250
Bovine Serum Albumin (BSA)	Sigma-Aldrich	A7906
Cell Trace Violet	ThermoFisher	C34571
Cell Tracker Deep Red	ThermoFisher	C34565
CFSE	ThermoFisher	C1157
Collagenase IV	Worthington biochemical	LS004188
cOmplete Protease Inhibitors cocktail	Roche	4693132001
CountBright Absolute Counting Beads	ThermoFisher	C36950
Dibutyl phtalate	Sigma-Aldrich	524980
Dnase I	Applichem	A3778
EDTA	Promega	V4231
Fetal bovine serumSerum (FBS)	Gibco	10270-106
Fluorescein isothiocyanate (FITC)	Sigma-Aldrich	F7250
Formaldehyde	ThermoFisher Scientific	J60401.AP
Goat serum	Sigma	G9023
GolgiPlug	BD Biosciences	555029
HAV antigen	Biozol	MBS318674
IL-2	Biolegend	575406
L-glutamine	Gibco	25030-024
Lipolysaccharides (LPS)	Sigma-Aldrich	L4516
Methanol	Fisher Scientific	M/4000/17
Optimal Cutting Temperature (OCT)	Tissue-Tek	4583
PBS	Sigma-Aldrich	D8537
PBS with calcium and magnesium	Sigma-Aldrich	D8662
Penicillin/Streptomycin	Gibco	15140-122
PowerUp SYBR Green	Applied Biosystems	A25776
Recombinant GM-CSF	Peprotech	315-03
RPMI	Gibco	31870-025
Sodium pyruvate	Gibco	11360-039
SYBR Green Master Mix	Roche	4707516001
T-PER medium	ThermoFisher	78510
Triton X-100	Sigma-Aldrich	X100
Trizol	Invitrogen	15596018
Tween-20	Applichem	A4974
β-mercaptoethanol	Gibco	31350-010

Commercial kits		
**Name**	**Company**	**Catalog #**
Direct-zol RNA MiniPrep kit	Zymo Research	R2052
EasySep™ Mouse $CD4^+$ T cell Isolation	Stem Cell Technologies	19852
EasySep™ Mouse $CD8^+$ T cell Isolation	Stem Cell Technologies	19853
Foxp3/Transcription Factor Staining buffer set	eBiosciences	00-5523-00
High-capacity RNA-to-cDNA	Applied Biosystems	4368814
Pierce BCA Protein Assay	ThermoFisher	23227

Supplementary Table 4. List of antibodies used in the different experiments

Flow cytometry antibodies					
**Antigen**	**Fluorophore**	**Clone**	**Company**	**Catalog #**	**Dilution**
CD3	AF700	17A2	eBiosciences	56-0032-82	1:50
CD3	PE/Cy7	17A2	Biolegend	100219	1:200
CD3	PE/DZL594	17A2	Biolegend	100246	1:200
CD3e	BV421	145-2C11	Biolegend	100336	1:100 - 1:200
CD3e	BUV395	145-2C11	BD bioscience	565992	1:200
CD4	APC	GK1.5	Biolegend	100412	1:100 - 1:400
CD4	BV480	GK1.5	BD bioscience	746475	1:200
CD4	BV650	RM4-5	BD bioscience	563747	1:200
CD4	BV711	GK1.5	Biolegend	100447	1:100 - 1:200
CD8a	APC/Cy7	53-6.7	Biolegend	100713	1:100
CD8a	BV605	53-6.7	BD bioscience	563152	1:200
CD8a	BV785	53-6.7	Biolegend	100750	1:100 - 1:200
CD8a	PE/Cy7	53-6.7	Biolegend	100722	1:400
CD11b	BV480	M1/70	BD bioscience	566149	1:200
CD11c	APC/Cy7	N418	Biolegend	117323	1:200
CD11c	BUV737	HL3	BD bioscience	612796	1:100 - 1:200
CD16/32	Purified	93	Biolegend	101302	1:50
CD19	BB700	1D3	BD bioscience	566411	1:200
CD25	Alexa Fluor 488	PC61	Biolegend	102017	1:100
CD25	BV480	PC61	BD bioscience	566202	1:100
CD40	PerCP-eFluor710	1C10	eBiosciences	46-0401-82	1:100
CD44	BUV737	IM7	BD bioscience	612799	1:200
CD45	BUV395	30-F11	BD bioscience	564279	1:200
CD45	BUV737	30-F11	BD bioscience	748371	1:200
CD45R/B220	BV650	RA3-6B2	BD bioscience	563893	1:200
CD45R/B220	PE	RA3-6B2	Biolegend	103208	1:100
CD45R/B220	PE/Cy7	RA3-6B2	Biolegend	103222	1:100
CD69	BUV737	H1.2F3	BD bioscience	612793	1:200
CD69	SB600	H1.2F3	eBiosciences	63-0691-82	1:100
CD80	PE/Cy5	16-10A1	Biolegend	104712	1:100
CD86	BUV395	GL1	BD bioscience	564199	1:100
CD95	APC-R700	Jo2	BD bioscience	565130	1:200
CD103	BV421	2E7	Biolegend	121422	1:100
CD138	APC	281-2	Biolegend	142505	1:200
CD185	PE/Cy7	L138D7	Biolegend	145516	1:200
CD197	APC-eFluor780	4B12	eBiosciences	47-1971-82	1:50
CD197	BV786	4B12	BD bioscience	564355	1:50
CD205	PE/Cy7	205yekta	eBiosciences	25-2051-42	1:100
CD279	FITC	29F.1A12	Biolegend	135213	1:200
CD326	Alexa Fluor 647	G8.8	Biolegend	118212	1:100
GL7	BV421	GL7	BD bioscience	562967	1:200
Granzyme B	PE	QA16A02	Biolegend	372207	1:200
IFN-γ	BV785	XMG1.2	Biolegend	505837	1:200
IL-2	Alexa Fluor 488	JES6-5H4	Biolegend	503837	1:200
IL-4	BV711	11B11	Biolegend	504133	1:200
IL-17A	BV421	C11-18H10.	Biolegend	506925	1:200
Ki67	PE	16A8	Biolegend	652404	1:200
MHCII	PE/Cy5	M5/114.15.2	Biolegend	107611	1:3000
MHCII	BV650	M5/114.15.2	Biolegend	107641	1:100 - 1:10000
NK1.1	PE/Cy5	PK136	Biolegend	108715	1:200
TNF	Alexa Fluor 647	MP6-XT22	Biolegend	506314	1:200
DAPI	-	-	Biolegend	422801	3 µM
DRAQ7	-	-	Biolegend	424001	2 µM
Fixable Viability dye	eFluor™ 780	-	eBiosciences	65-0865-18	1:1000
Propidium Iodide	-	-	Invitrogen	P3566	1.7 - 5 µg/ml

Other antibodies						
**Antigen**	**Fluorophore**	**Clone**	**Company**	**Catalog #**	**Dilution**	**Use**
CD11a	-	M17/4	BioXCell	BE0006	100 ug/mouse	*In vivo* blocking
CD49d	-	PS/2	BioXCell	BE0071	100 ug/mouse	*In vivo* blocking
ICAM-1	-	YN1/1.7.4	BioXCell	BE0020-1	100 ug/mouse	*In vivo* blocking
TNF	-	XT3.11	BioXCell	BE0058	100 or 500 ug/mouse	*In vivo* blocking
Rat IgG1 isotype	-	HRPN	BioXCell	BE0088	50 ug/mouse	*In vivo* blocking
Rat IgG2a,κ isotype	-	2A3	BioXCell	BE0089	50 ug/mouse	*In vivo* blocking
Rat IgG2b,κ isotype	-	LTF-2	BioXCell	BE0090	50 ug/mouse	*In vivo* blocking
BMAL1	-	D2L7G	Cell Signalling Techology	14020S	1:500	ChIP
IgG	-	-	Abcam	ab171870	1:500	ChIP
IgG	HRP	-	Invitrogen	A16072	1:1000	ELISA
CD3	-	145-2C11	Invitrogen	16-0031-85	2 ug/ml	Proliferation assay
CD28	-	37.51	Invitrogen	16-0281-82	2 ug/ml	Proliferation assay
CD31	APC	390	Biolegend	102410	1:100	Imaging
ICAM-1	PE	YN1/1.7.4	Biolegend	116108	1:100	Imaging
Rat IgG2b,κ isotype	PE	RTK4530	Biolegend	400608	1:100	Imaging

Supplementary Note 1. Mathematical model development for incorporating multiple rhythmic elements in generation of immune response

Mathematical model describing interaction of multiple rhythms (ID4)

With rhythmicity detected in the individual components of lymphocyte and dendritic cell (DC) homing to individual lymph nodes (LNs), as well as in cell proliferation and time-dependent cell velocity dynamics within the LN, we developed a mathematical model to determine how the interaction of the different rhythmic components shapes immune responses and leads to maintained rhythmicity. To this end, we combined the individual dynamics shown in Eqs. (1) – (4) before. We additionally assume that a concentration of activated T cells, T_A, is formed based on the interaction of lymphocytes and DC in the LN at an activation rate σ. This activation is shaped by a factor v_{ZT}, which accounts for the different velocities of T cells dependent on ZT with v_{ZT}=1 for ZT=7. Activated T cells start to proliferate, T_P, at rate ρ, which is additionally shaped by the rhythmic division index, *DI*(t) as defined within Eq. (4). To prevent unlimited LN expansion, a carrying capacity *N* is assumed that defines the maximal number of cells in a lymph node. Therefore, infiltration of unactivated T cells, *X(t)*, as well as proliferation of activated T cells is assumed to saturate with increasing LN expansion. In addition, *DC* are lost from the LN at a rate γ. A sketch of the model is provided in **Figure 4b** of the main manuscript. The whole model is then defined by:

$$\frac{dX}{dt} = A\left(1+f_1(\omega,\phi_h,t)\right)\left(1-\frac{X+T_A+T_P}{N}\right) - d\left(1+\sin(\omega(t+\phi_e))\right)X \quad (5)$$

$$\frac{DC_E}{dt} = -\alpha\left(1+\sin(\omega(t+\phi_{h,DC}))\right)DC_E - \mu DC_E \quad (6)$$

$$\frac{DC_I}{dt} = \alpha\left(1+\sin(\omega(t+\phi_{h,DC}))\right)DC_E + \mu DC_E - \gamma DC_I \quad (7)$$

$$\frac{dT_A}{dt} = \nu_{ZT}\sigma X DC_I \quad (8)$$

$$\frac{dT_P}{dt} = \rho DI(t)\left(1-\frac{X+T_A+T_P}{N}\right)(T_A+T_P) \quad (9)$$

$$-d\left(1+\sin(\omega(t+\phi_e))\right)\left(\frac{1}{1+\lambda(X+T_A+T_P)}\right)T_P \quad (10)$$

with

$$f_1(\omega,\phi_h,t) = \begin{cases} A\left(1+\sin(\omega(t+\phi_h))\right) & \text{if } t < t_{ZT} \\ \max\left[A\left(1+\sin(\omega(t+\phi_h))\right), A\left(1+\sin(\omega(t_{ZT}+\phi_h))\right)\right] & \text{if } t \geq t_{ZT} \end{cases}$$

In comparison to the description of lymphocyte homing and egress in Eq.(1), we found that two modifications for the model were necessary to describe the observed LN expansion, as well as maintaining rhythmicity throughout time. These modifications account for altered lymphocyte recruitment and egress dynamics due to T-DC interactions within the lymph node. The function $f_1(\omega,\phi_h,t)$ accounts for the fact that T-DC interactions facilitate lymphocyte recruitment (e.g. due to inflammation) by ensuring lymphocyte increase to be not smaller than the influx generated at the time of injection of DC, i.e., t_{ZT}. Furthermore, a delayed egress of activated lymphocytes within in the lymph node is assumed, with the egress rate *d* reduced in a density-dependent manner. Hereby, the parameter λ denotes a scaling factor.

The model was parameterized using the estimates of the individual components obtained from ID1-ID3 (see **Table S1**). In addition, the velocity factor v_{ZT} was calculated based on the ratio of the mean velocities measured (**Figure 1f**). Furthermore, we assumed an activation rate of σ = 0.011 h^{-1} and a cell proliferation rate of ρ = 0.03 h^{-1}, corresponding to an average division period of ~33 h, to match reasonable cell numbers within LN. The average dwell time of DC within the LN contributing to T cell activation, $1/\gamma$, was set to 50 h, and the scaling factor regulating the efflux of activated T cells was set to λ = 10^{-3} based on the analyses of previous data sets. All individual parameters used for simulation are given in **Table S1**.

The different skin draining lymph nodes, i.e. axillary, inguinal and superficial cervical LN, were simulated individually and the average dynamics across all lymph nodes was calculated to compare LN expansion using different ZT time points for DC injection. DC were assumed to migrate to the LN for a time period of 1h after injection. We tested different model assumptions with the mentioned feedback dynamics of T-DC interactions on lymphocyte recruitment and egress being able to explain the observed maintenance of rhythmicity within the immune response at later time points.
