# Supplementary material for: Influence of circadian clocks on adaptive immunity and vaccination responses
Source: Nat Commun. 2023 Jan 30;14:476. doi: 10.1038/s41467-023-35979-2 (PMC9885059; doi:10.1038/s41467-023-35979-2)
Supplement: Supplementary file 2 — Reporting Summary [file 41467_2023_35979_MOESM2_ESM.pdf]

nature portfolio

Corresponding author(s): Prof. Christoph Scheiermann

Last updated by author(s): Dec 5, 2022

Reporting Summary

Nature Portfolio wishes to improve the reproducibility of the work that we publish. This form provides structure for consistency and transparency in reporting. For further information on Nature Portfolio policies, see our Editorial Policies and the Editorial Policy Checklist.

Statistics

For all statistical analyses, confirm that the following items are present in the figure legend, table legend, main text, or Methods section.

n/a	Confirmed	
☐	☒	The exact sample size (*n*) for each experimental group/condition, given as a discrete number and unit of measurement
☐	☒	A statement on whether measurements were taken from distinct samples or whether the same sample was measured repeatedly
☐	☒	The statistical test(s) used AND whether they are one- or two-sided<br>*Only common tests should be described solely by name; describe more complex techniques in the Methods section.*
☒	☐	A description of all covariates tested
☐	☒	A description of any assumptions or corrections, such as tests of normality and adjustment for multiple comparisons
☐	☒	A full description of the statistical parameters including central tendency (e.g. means) or other basic estimates (e.g. regression coefficient) AND variation (e.g. standard deviation) or associated estimates of uncertainty (e.g. confidence intervals)
☐	☒	For null hypothesis testing, the test statistic (e.g. *F*, *t*, *r*) with confidence intervals, effect sizes, degrees of freedom and *P* value noted<br>*Give P values as exact values whenever suitable.*
☒	☐	For Bayesian analysis, information on the choice of priors and Markov chain Monte Carlo settings
☒	☐	For hierarchical and complex designs, identification of the appropriate level for tests and full reporting of outcomes
☒	☐	Estimates of effect sizes (e.g. Cohen's *d*, Pearson's *r*), indicating how they were calculated

Our web collection on statistics for biologists contains articles on many of the points above.

Software and code

Policy information about availability of computer code

Data collection	BD FACSDiva 8, Kaluza (Beckman Coulter), StepOne v2.3, SlideBook 6, Leica Application Suite, Softmax Pro
Data analysis	GraphPad Prism v9, ImageJ v.1.51.n and ImageJ plugin TrackMate, FlowJo v10, MaxQuant v.1.5.6.2i, Perseus v.1.5.5.5, R, Excel

For manuscripts utilizing custom algorithms or software that are central to the research but not yet described in published literature, software must be made available to editors and reviewers. We strongly encourage code deposition in a community repository (e.g. GitHub). See the Nature Portfolio guidelines for submitting code & software for further information.

Data

Policy information about availability of data

All manuscripts must include a data availability statement. This statement should provide the following information, where applicable:

- Accession codes, unique identifiers, or web links for publicly available datasets
- A description of any restrictions on data availability
- For clinical datasets or third party data, please ensure that the statement adheres to our policy

All data generated in this study have been deposited in the Yareta database under accession code https://doi.org/10.26037/yareta:hs6p7nzpkfhrlavgdopkqhafpm.

Human research participants

Policy information about studies involving human research participants and Sex and Gender in Research.

Reporting on sex and gender	N/A
Population characteristics	N/A
Recruitment	N/A
Ethics oversight	N/A

Note that full information on the approval of the study protocol must also be provided in the manuscript.

Field-specific reporting

Please select the one below that is the best fit for your research. If you are not sure, read the appropriate sections before making your selection.

- [x] Life sciences
- [ ] Behavioural & social sciences
- [ ] Ecological, evolutionary & environmental sciences

For a reference copy of the document with all sections, see nature.com/documents/nr-reporting-summary-flat.pdf

Life sciences study design

All studies must disclose on these points even when the disclosure is negative.

Sample size	Sample size was not determined prior to the experiments. Based on our prior experience, we estimated that 3 mice per group would be enough to have statistically difference at least. When a strong tendency or high division were observed, sample size will be increased.
Data exclusions	Data was not excluded, unless suggested via statistical testing (GraphPad, Identitication of outliers, ROUT method, Q=1%) or in cases where additional biological reasons indicated a compromised sample (e.g. injured animal)
Replication	Experiments were designed with a minimum of 3 biological replicates and analyzed with 2 technical replicates (e.g. qPCR, cell culture) which were successful
Randomization	Mice were randomly allocated into different experimental groups
Blinding	Wherever possible investigators were blinded to group allocation. It was not possible to blind investigators to time-of-day or duration after treatment during sample collection since animals has to be kept in the proper lighting regimen as long as possible. However, investigators were blind to genotype and/or treatment where applicable.

Reporting for specific materials, systems and methods

We require information from authors about some types of materials, experimental systems and methods used in many studies. Here, indicate whether each material, system or method listed is relevant to your study. If you are not sure if a list item applies to your research, read the appropriate section before selecting a response.

Materials & experimental systems

n/a	Involved in the study	
[ ]	[x]	Antibodies
[x]	[ ]	Eukaryotic cell lines
[x]	[ ]	Palaeontology and archaeology
[ ]	[x]	Animals and other organisms
[x]	[ ]	Clinical data
[x]	[ ]	Dual use research of concern

Methods

n/a	Involved in the study	
[x]	[ ]	ChIP-seq
[ ]	[x]	Flow cytometry
[x]	[ ]	MRI-based neuroimaging

Antibodies

Antibodies used	The following anti-mouse antibodies were used for immunostaining: CD3 AF700 17A2 eBiosciences 56-0032-82<br>CD3 (PE/Cy7, clone 17A2, Biolegend, 100219); CD3 (PE/DZL594, clone 17A2, Biolegend, 100246); CD3e (BV421, clone 145-2C11, Biolegend, 100336); CD3e (BUV395, clone 145-2C11, BD bioscience, 565992); CD4 (APC, clone GK1.5, Biolegend, 100412); CD4 (BV480, clone GK1.5, BD bioscience, 746475); CD4 (BV650, clone RM4-5, BD bioscience, 563747); CD4 (BV711, clone GK1.5,

Biolegend, 100447); CD8a (APC/Cy7, clone 53-6.7, Biolegend, 100713); CD8a (BV605, clone 53-6.7, BD bioscience, 563152); CD8a (BV785, clone 53-6.7, Biolegend, 100750); CD8a (PE/Cy7, clone 53-6.7, Biolegend, 100722); CD11b (BV480, clone M1/70, BD bioscience, 566149); CD11c (APC/Cy7, clone N418, Biolegend, 117323); CD11c (BUV737, clone HL3, BD bioscience, 612796); CD16/32 (Purified, clone 93, Biolegend, 101302); CD19 (BB700, clone 1D3, BD bioscience, 566411); CD25 (Alexa Fluor 488, clone PC61, Biolegend, 102017); CD25 (BV480, clone PC61, BD bioscience, 566202); CD40 (PerCP-eFluor710, clone 1C10, eBiosciences, 46-0401-82); CD44 (BUV737, clone IM7, BD bioscience, 612799); CD45 (BUV395, clone 30-F11, BD bioscience, 564279); CD45 (BUV737, clone 30-F11, BD bioscience, 748371); CD45R/B220 (BV650, clone RA3-6B2, BD bioscience, 563893); CD45R/B220 (PE, clone RA3-6B2, Biolegend, 103208); CD45R/B220 (PE/Cy7, clone RA3-6B2, Biolegend, 103222); CD69 (BUV737, clone H1.2F3, BD bioscience, 612793); CD69 (SB600, clone H1.2F3, eBiosciences, 63-0691-82); CD80 (PE/Cy5, clone 16-10A1, Biolegend, 104712); CD86 (BUV395, clone GL1, BD bioscience, 564199); CD95 (APC-R700, clone Jo2, BD bioscience, 565130); CD103 (BV421, clone 2E7, Biolegend, 121422); CD138 (APC, clone 281-2, Biolegend, 142505); CD185 (PE/Cy7, clone L138D7, Biolegend, 145516); CD197 (APC-eFluor780, clone 4B12, eBiosciences, 47-1971-82); CD197 (BV786, clone 4B12, BD bioscience, 564355); CD205 (PE/Cy7, clone 205yekta, eBiosciences, 25-2051-42); CD279 (FITC, clone 29F.1A12, Biolegend, 135213); CD326 (Alexa Fluor 647, clone G8.8, Biolegend, 118212); GL7 (BV421, clone GL7, BD bioscience, 562967); Granzyme B (PE, clone QA16A02, Biolegend, 372207); IFN-γ (BV785, clone XMG1.2, Biolegend, 505837); IL-2 (Alexa Fluor 488, clone JES6-5H4, Biolegend, 503837); IL-4 (BV711, clone 11B11, Biolegend, 504133); IL-17A (BV421, clone TC11-18H10.1, Biolegend, 506925); Ki67 (PE, clone 16A8, Biolegend, 652404); MHCII (PE/Cy5, clone M5/114.15.2, Biolegend, 107611); MHCII (BV650, clone M5/114.15.2, Biolegend, 107641); NK1.1 (PE/Cy5, clone PK136, Biolegend, 108715); TNF (Alexa Fluor 647, clone MP6-XT22, Biolegend, 506314); DAPI (Biolegend, 422801); DRAQ7 (Biolegend, 424001); Fixable Viability dye eFluor™ 780 (eBiosciences, 65-0865-18); Propidium Iodide (Invitrogen, P3566).

For in vivo treatment, anti-mouse CD11a (clone M17/4, BioXCell, BE0006); CD49d (clone PS/2, BioXCell, BE0071); ICAM-1 (clone YN1/1.7.4, BioXCell, BE0020-1); TNF (clone XT3.11, BioXCell, BE0058); Rat IgG1 isotype (clone HRPN, BioXCell, BE0088); Rat IgG2a,κ isotype (clone 2A3, BioXCell, BE0089); Rat IgG2b,κ isotype (clone LTF-2, BioXCell, BE0090).

For ChIP experiment: anti-mouse/human BMAL1 (clone D2L7G, Cell Signalling Techology, 14020S); IgG (Abcam, ab171870).

For ELISA detection: anti-mouse IgG (Invitrogen, A16072).

For proliferation assays: anti-mouse CD31 APC 390 Biolegend 102410
ICAM-1 PE YN1/1.7.4 Biolegend 116108
Rat IgG2b,κ isotype PE RTK4530 Biolegend 400608
CD3 (clone 145-2C11, Invitrogen, 16-0031-85); CD28 (clone 37.51, Invitrogen, 16-0281-82).

For imaging experiments: anti-mouse CD31 (APC, clone 390, Biolegend, 102410); ICAM-1 (PE, clone YN1/1.7.4, Biolegend, 116108); Rat IgG2b,κ isotype (PE, clone RTK4530, Biolegend, 400608).

Validation	Primary antibody has been validated by the manufacturer for the specific species. All neutralization antibodies used were taken from publications that have validated the antibodies prior to this study.

Animals and other research organisms

Policy information about studies involving animals; ARRIVE guidelines recommended for reporting animal research, and Sex and Gender in Research

Laboratory animals	C57BL/6N wild-type (WT) mice (Mus musculus, mixed genders, 7-12 weeks old) were purchased from Charles River. The following transgenic mice were cross-bred at ENVIGO to yield T cell-specific (BMAL1ΔTcell, Mus musculus, C57BL/6 background, mixed genders, 7-12 weeks old) and inducible endothelial cell specific (BMAL1ΔEC Mus musculus, C57BL/6 background, mixed genders, 5-12 weeks old) Bmal1-deficient mice: Bmal1flox/flox, Cd4Cre (both purchased from Jackson labs), Cdh5CreERT2 (gift from Dr. Ralf Adams, Max-Planck-Institute for Molecular Biomedicine Münster, Germany).
Wild animals	The study did not involve wild animals.
Reporting on sex	This study used mixed genders animals
Field-collected samples	This study did not involve field-collected samples.
Ethics oversight	All animal procedures and experiments were performed in strict accordance with all mandatory guidelines (EU and Swiss directives on the protection of animals used for scientific purposes), approved by the ministry of animal welfare of the region of Oberbayern and performed in accordance with the German law of animal welfare or approved and performed in accordance with the guidelines of the animal research committee of Geneva (Commission Cantonale pour les Expériences sur les Animaux (CCEA) and the Office fédéral de la santé alimentaire et des affaires vétérinaires (OSAV)).

Note that full information on the approval of the study protocol must also be provided in the manuscript.

Flow Cytometry

Plots

Confirm that:

- [x] The axis labels state the marker and fluorochrome used (e.g. CD4-FITC).
- [x] The axis scales are clearly visible. Include numbers along axes only for bottom left plot of group (a 'group' is an analysis of identical markers).
- [x] All plots are contour plots with outliers or pseudocolor plots.
- [x] A numerical value for number of cells or percentage (with statistics) is provided.

Methodology

Sample preparation	To obtain single cell suspensions, organs were first finely minced with scissors and incubated in 1 ml digestion mix (PBS with Ca2+ and Mg2+, 1 mg/ml collagenase IV and 0.2 mg/ml DNase I) for 30 min at 37°C under constant agitation. For analysis of germinal centers following vaccination, digestion was unnecessary, and this step was omitted. Samples were processed through 70 µm cell strainers (Corning) with an excess volume of PEB (PBS with 1% BSA and 2mM EDTA) and centrifuged at 300 g for 5 min. For spleen samples, cell pellets were resuspended in 5 ml RBC lysis buffer (155mM NH4Cl, 0.1mM KHCO3, 0.1mM EDTA in ddH2O) and incubated for 5 min at room temperature. 5ml PEB was added to stop the lysis reaction and samples were centrifuged again. Cell pellets were resuspended in PEB prior to staining. Spleen samples were counted and 2 x 106 cells were taken for staining to ensure saturating conditions. LN samples were stained in their entirety with a small aliquot taken for counting during staining on a Coulter Counter (Z2, Beckman Coulter; Countess II, Invitrogen).<br>For all staining panels, an initial blocking step was performed to reduce non-specific binding. Cells were incubated with 10 µg/ml purified rat anti-mouse CD16/32 antibody (101302, Biolegend) in PEB at room temperature for 5-15 minutes. Following this incubation, an equivalent volume of 2x concentrated antibody staining mix was added directly to the cell suspension and samples were incubated for a further 30 minutes at 4 °C. Cells were washed and resuspended in 300 µl PEB with assay-dependent viability dye (DAPI, Biolegend, 3 µM; Propidium Iodide, Invitrogen, 1.7 µg/ml; or DRAQ7, Biolegend, 2 µM) and characterised using a 10-colour Gallios (Beckman Coulter) or 18-colour BD LSR Fortessa (BD Biosciences) flow cytometer.
Instrument	10-colour Gallios (Beckman Coulter); 18-colour BD LSR Fortessa (BD Biosciences) flow cytometers
Software	Kaluza (Beckman Coulter), FACSDiva 8 (BD Biosciences) and FlowJo 10 (TreeStar) softwares
Cell population abundance	This study did not involve cell sorting.
Gating strategy	Debris and doublets were excluded based on forward and side scatter profiles, and viability dye was used to remove dead cells. Fluorescence minus one (FMO) controls were used to define gate positions.

- [x] Tick this box to confirm that a figure exemplifying the gating strategy is provided in the Supplementary Information.
